# Xrn2 accelerates termination by RNA polymerase II, which is underpinned by CPSF73 activity

**DOI:** 10.1101/gad.308528.117

**Published:** 2018-01-15

**Authors:** Joshua D. Eaton, Lee Davidson, David L.V. Bauer, Toyoaki Natsume, Masato T. Kanemaki, Steven West

**Affiliations:** 1 The Living Systems Institute, University of Exeter, Exeter EX4 4QD, United Kingdom;; 2 Department of Molecular Biology and Biotechnology, University of Sheffield, Sheffield S10 2TN, United Kingdom;; 3 Sir William Dunn School of Pathology, University of Oxford, Oxford OX1 3RE, United Kingdom;; 4 Division of Molecular Cell Engineering, National Institute of Genetics, Research Organization of Information and Systems (ROIS), Mishima, Shizuoka 411-8540, Japan;; 5 Department of Genetics, Graduate University for Advanced Studies (SOKENDAI), Mishima, Shizuoka 411-8540, Japan

**Keywords:** Xrn2, transcriptional termination, CPSF73, torpedo, allosteric, RNA polymerase II

## Abstract

Termination is a ubiquitous phase in every transcription cycle but is incompletely understood and a subject of debate. We used gene editing as a new approach to address its mechanism through engineered conditional depletion of the 5′ → 3′ exonuclease Xrn2 or the polyadenylation signal (PAS) endonuclease CPSF73 (cleavage and polyadenylation specificity factor 73). The ability to rapidly control Xrn2 reveals a clear and general role for it in cotranscriptional degradation of 3′ flanking region RNA and transcriptional termination. This defect is characterized genome-wide at high resolution using mammalian native elongating transcript sequencing (mNET-seq). An Xrn2 effect on termination requires prior RNA cleavage, and we provide evidence for this by showing that catalytically inactive CPSF73 cannot restore termination to cells lacking functional CPSF73. Notably, Xrn2 plays no significant role in either Histone or small nuclear RNA (snRNA) gene termination even though both RNA classes undergo 3′ end cleavage. In sum, efficient termination on most protein-coding genes involves CPSF73-mediated RNA cleavage and cotranscriptional degradation of polymerase-associated RNA by Xrn2. However, as CPSF73 loss caused more extensive readthrough transcription than Xrn2 elimination, it likely plays a more underpinning role in termination.

## Abstract (precis)

In this study, Eaton et al. use a new gene-editing approach to delineate the mechanism by which RNA polymerase II terminates. They generated human cell lines from which the 5′-to-3′ exoribonuclease Xrn2 or the poly(A) signal endoribonuclease CPSF73 can be rapidly controlled and show that efficient termination on most protein-coding genes involves CPSF73-mediated RNA cleavage and cotranscriptional degradation of polymerase-associated RNA by Xrn2.

Transcriptional termination can be defined as the cessation of RNA polymerization and dissolution of the ternary complex of RNA polymerase II (Pol II), DNA, and RNA. Termination is a biologically important process, as it prevents transcriptional interference of genes and ensures that polymerases are available for new rounds of gene expression. Despite the fact that all transcription ends this way, it is perhaps the least understood phase in the cycle. A polyadenylation signal (PAS) is a prerequisite for termination, and mutations within it were shown decades ago to cause extended transcriptional readthrough (Whitelaw and Proudfoot 1986; Connelly and Manley 1988). Two models, the allosteric and torpedo, have since framed efforts to understand PAS-dependent termination (Porrua and Libri 2015; Proudfoot 2016). In the allosteric mechanism, transcription of a PAS causes a change in Pol II structure or alters the composition of the elongation complex to promote termination. In the torpedo model, RNA cleavage generates a Pol II-associated RNA substrate for 5′ → 3′ degradation that triggers termination by pursuing and catching the polymerase (Connelly and Manley 1988; Proudfoot 1989). Multiple studies provide support for both models, with the actual mechanism likely to incorporate aspects of each. However, their relative contributions are debated due to different results obtained in a variety of experimental systems (Libri 2015).

Early support for the torpedo model came from observations that depletion of the nuclear 5′ → 3′ exonuclease Xrn2 caused termination defects on transfected plasmids (West et al. 2004). Its homolog, Rat1, was simultaneously found to promote termination more widely in budding yeast (Kim et al. 2004), with recent transcriptome-wide analysis supporting this finding (Baejen et al. 2017). The broader role of Xrn2 in human cells has been less clear. RNAi of Xrn2 showed no general function in termination at the 3′ ends of protein-coding genes (Nojima et al. 2015), but a significant effect was later observed upon concurrent expression of catalytically dead Xrn2 (Fong et al. 2015). It is likely that the inactive protein binds Xrn2 substrates and blocks their degradation by the diminished levels of endogenous Xrn2. As such, RNAi may not always reveal the complete set of functions for some proteins.

Rat1 was shown to promote the recruitment of some polyadenylation factors to budding yeast genes and so may sometimes affect termination indirectly through impacting PAS function (Luo et al. 2006). In this instance, cotranscriptional degradation of PAS-cleaved RNA was insufficient to cause termination on some genes, highlighting the possibility that RNA degradation may not always release polymerase (Luo et al. 2006). Even so, catalytically inactive Rat1 does not support termination on other yeast genes, and Rat1, Xrn1, and Xrn2 can all dissociate Pol II from DNA in purified systems (Kim et al. 2004; Park et al. 2015). In *Caenorhabditis elegans*, Xrn2 depletion does not affect termination on the majority of protein-coding genes, suggesting that the torpedo mechanism is less widely used in that organism (Miki et al. 2017).

To understand the extent to which the allosteric and torpedo models explain the termination mechanism, it is important to distinguish the role of PAS recognition from PAS cleavage, which is difficult to do in vivo. A human PAS is recognized by several multisubunit complexes that bind to its AAUAAA hexamer and downstream G/U-rich motif (Proudfoot 2012). AAUAAA is recognized by the CPSF30 and WDR33 subunits of cleavage and polyadenylation specificity factor (CPSF), with endonuclease activity provided by CPSF73 (Mandel et al. 2006; Shi et al. 2009; Chan et al. 2014; Schonemann et al. 2014). Although CPSF73 was identified as the nuclease over a decade ago (Mandel et al. 2006), its function in termination is not fully characterized. This issue has been tackled using in vitro systems competent for transcription and RNA processing, which revealed that a PAS can promote termination in the absence of cleavage (Zhang et al. 2015). While highlighting the capacity of PAS recognition to affect Pol II activity, it is unknown whether this mechanism promotes termination in cells.

Therefore, several aspects of termination in human cells are incompletely understood, especially in terms of their generality, and understanding of the process has lagged behind that of other model organisms. It is not known whether Xrn2 degrades PAS-cleaved RNA generally or whether this process is cotranscriptional, as was envisaged in the torpedo model. Possible effects of Xrn2 on PAS cleavage are also not established in a global manner. It is also unclear whether PAS cleavage is required for termination or whether polymerase release can be promoted by cleavage-independent factors, which is an issue that has an impact on the applicability of current models.

As RNAi approaches take days and since protein depletion is often incomplete, we adopted gene editing to engineer conditional depletion of Xrn2 or CPSF73 on faster time scales. This was used to show that Xrn2 degrades the 3′ product of PAS cleavage cotranscriptionally and promotes efficient termination genome-wide, which we mapped transcriptome-wide at high resolution. Importantly, we show that CPSF73 activity is required for efficient termination, confirming a primary mechanism in which PAS cleavage precedes degradation of polymerase-associated RNA. However, CPSF73 elimination causes stronger termination defects than the loss of Xrn2, suggesting that it might promote termination by additional mechanisms when the primary process fails.

## Results

### An auxin-inducible degron (AID) system for rapid Xrn2 depletion

**Figure 1.**
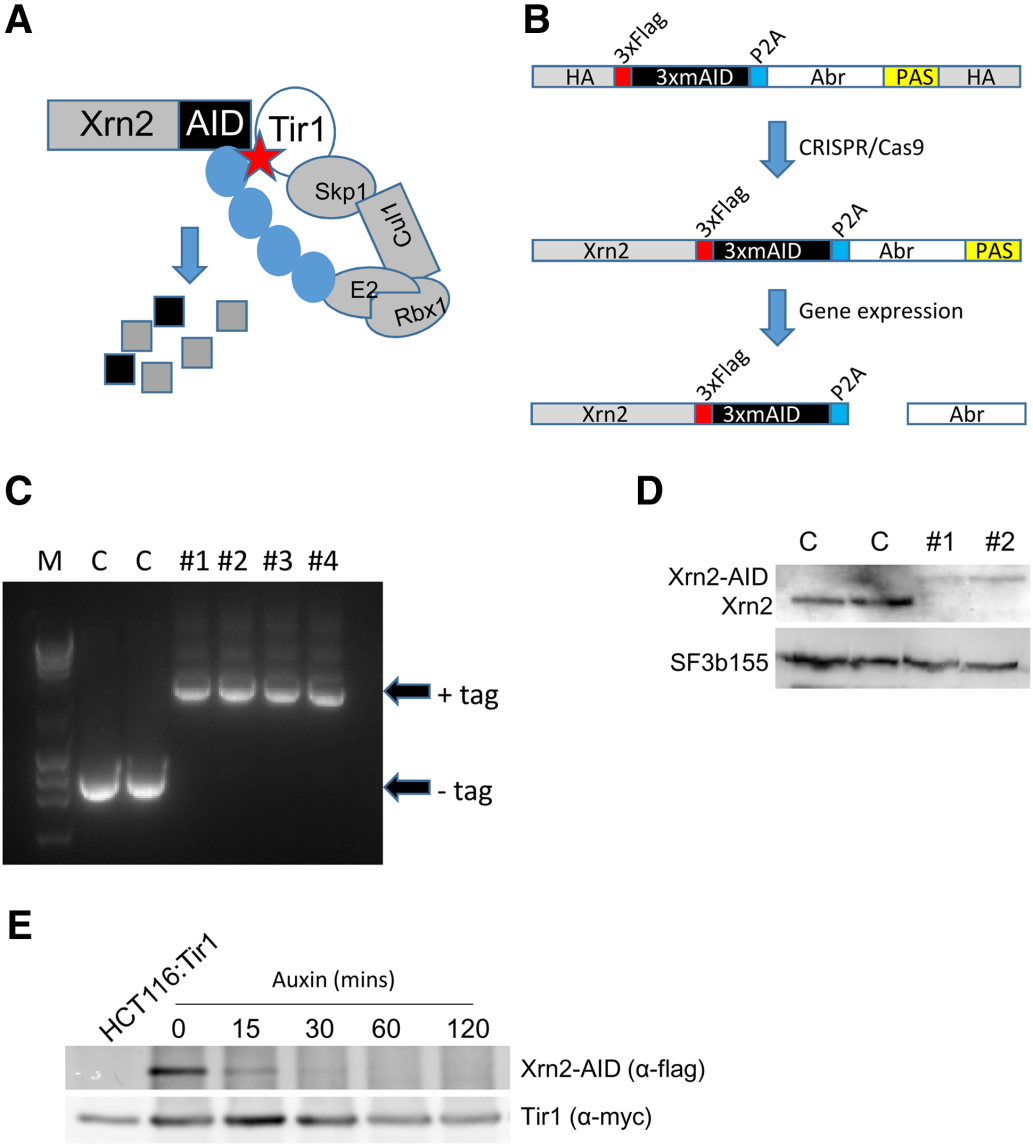
(*A*) Diagram showing the basis of auxin-dependent depletion of AID-tagged proteins. In the presence of auxin (star), Tir1 facilitates ubiquitination (blue circles) of the AID tag and rapid protein degradation. (*B*) Strategy for AID tagging of Xrn2. Homology arms (HAs) flanked repair cassettes containing 3× miniAID sequences, preceded by a Flag tag and separated from an antibiotic resistance gene (denoted as Abr and either Neo or Hyg) by a P2A cleavage site, with 3′ end processing driven by an SV40 PAS. (*C*) Diagnostic PCR of genomic DNA from antibiotic-resistant cell colonies following CRISPR gene editing. The presence of a tag increases the size of the PCR product compared with the smaller product derived from the unmodified gene. Homozygous modification is shown by the lack of unmodified product in the four drug-resistant colonies (#1–#4). (M) DNA marker. (*D*) Western blot confirmation of Xrn2 tagging. The *top* panel shows Xrn2 in two unmodified cell samples (C) and two gene-edited colonies (#1 and #2). Successful biallelic tagging is shown by the higher-molecular-weight species and the lack of native-sized Xrn2 in CRISPR-modified cells. SF3b155 was probed for as a loading control. (*E*) Time course of auxin addition on *XRN2-AID* cells. Xrn2-AID was detected by anti-Flag, and specificity is shown by the lack of product in Tir1 HCT116 cells, which are not modified at *XRN2*. Tir1 was probed for as a loading control via its myc tag.

To set up a system for rapid elimination of Xrn2, CRISPR/Cas9 was used to tag *XRN2* with an AID (Fig. 1A,B). AID-tagged proteins are degraded upon addition of indole-3-acetic acid (referred to here as auxin [IAA]) in a manner dependent on plant Tir1 protein (Nishimura et al. 2009; Natsume et al. 2016). HCT116 cells were chosen for this experiment due to their diploid nature. Cells expressing Tir1 were subjected to CRISPR/Cas9 genome editing using repair templates that incorporated three tandem miniAID degrons and hygromycin or neomycin selection markers (Kubota et al. 2013; Natsume et al. 2016). Selection markers were separated from the tag by a P2A sequence that was cleaved during translation (Kim et al. 2011). Transfection of these two constructs together with an *XRN2*-specific guide RNA expressing Cas9 plasmid yielded multiple resistant colonies, and homozygous modification was demonstrated by PCR (Fig. 1C).

Western blotting confirmed homozygous targeting in two selected positive clones, shown by the higher-molecular-weight Xrn2 and the absence of any signal at the size expected for native Xrn2 (Fig. 1D). It is notable that Xrn2-AID is present at lower levels than endogenous Xrn2, suggesting a destabilizing effect of the tag. Even so, *XRN2-AID* cells showed no growth defects (Supplemental Fig. 1A). Further RNA analyses performed throughout this study also showed that RNA degradation functions are virtually unimpaired in *XRN2-AID* cells.

To test Xrn2-AID depletion, Western blotting was performed over a time course of auxin addition (Fig. 1E). Xrn2-AID was detected through the Flag epitope present within the AID tag, with specificity shown by a lack of signal in unmodified HCT116 cells. Importantly, Xrn2-AID levels are reduced within 30 min of auxin treatment and were virtually undetectable after 1 h. As such, this system allows rapid and conditional depletion of Xrn2. The addition of auxin to the culture medium of *XRN2-AID* cells completely prevented cell colony formation, showing that Xrn2 is an essential protein (Supplemental Fig. 1B).

### Xrn2 plays a general role in the degradation of 3′ flanking region RNA

**Figure 2.**
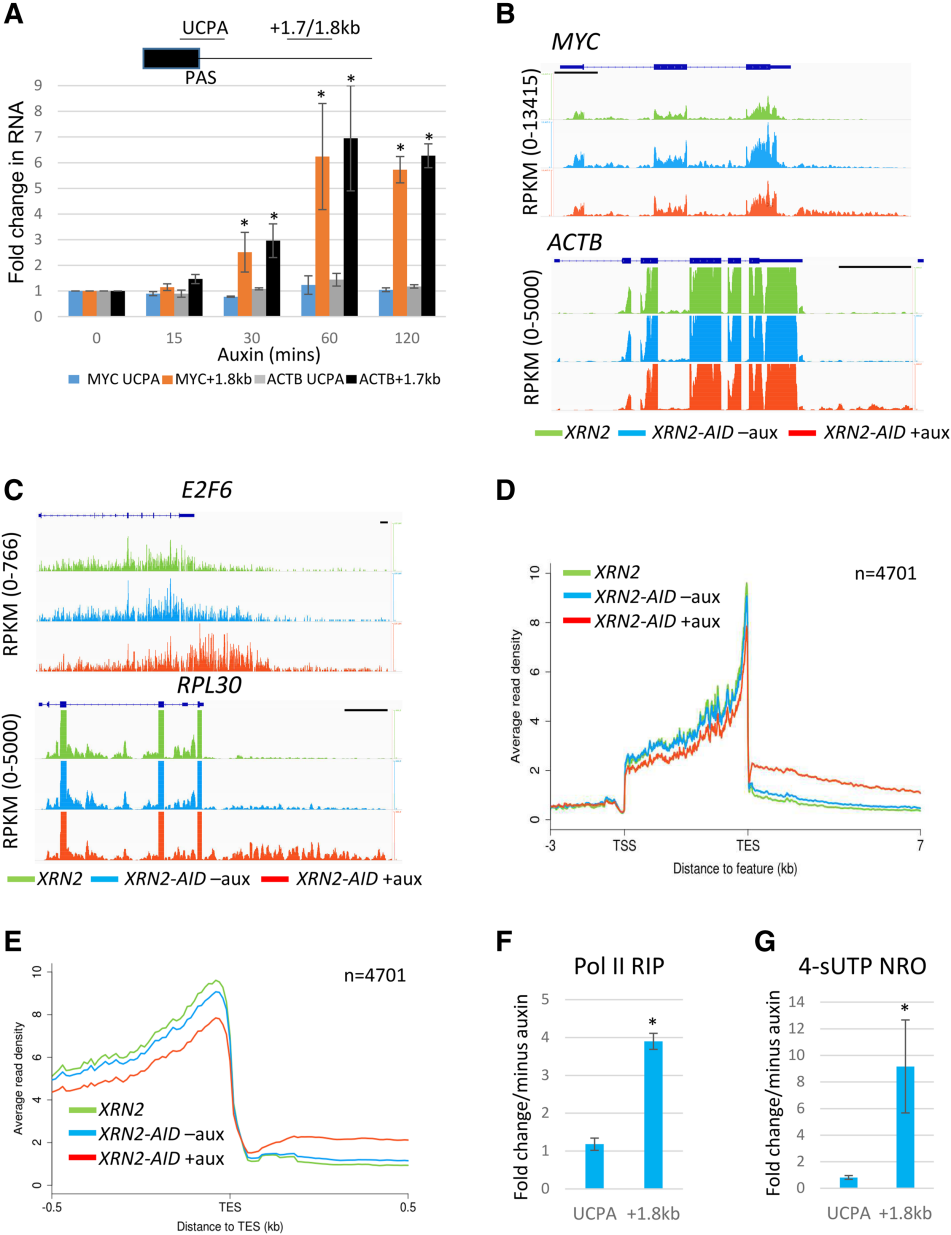
(*A*) qRT–PCR analysis of UCPA and 3′ flanking RNA from *MYC* and *ACTB* genes from total RNA during a time course of auxin addition. Values are plotted relative to those obtained at t0 after normalization to unspliced RNA levels from the respective genes. The diagram depicts the positions of UCPA amplicons and 3′ flank amplicons for both genes (+1.7 kb for *ACTB* and +1.8 kb for *MYC*). Asterisks denote *P* < 0.05 for changes relative to t0 in the absence of auxin. (*B*) Nuclear RNA sequencing (RNA-seq) traces of *MYC* and *ACTB* genes in samples obtained from *XRN2* unmodified cells and *XRN2-AID* cells treated with auxin for 1 h or untreated. The *Y*-axis shows RPKM (reads per kilobase transcript per million mapped reads). Bars, 1 kb. (*C*) As in *B* but showing *E2F6* and *RPL30* genes. (*D*) Metagene plots from nuclear RNA-seq on *XRN2* unmodified cells and *XRN2-AID* cells treated with auxin or untreated. The graph shows the region from 3 kb upstream of the transcription start site (TSS) up to 7 kb beyond the PAS (denoted as transcript end site [TES]). (*E*) A zoomed in view of ±0.5 kb of the TES from the same metagene presented in *D*. (*F*) Pol II RNA immunoprecipitation analysis of UCPA and 3′ flanking (+1.8 kb) RNA from *MYC* in cells depleted of Xrn2-AID (1 h of auxin treatment) or not. Quantitation is shown for +auxin samples relative to −auxin after normalizing to the level of unspliced *MYC* RNA. The asterisk denotes the difference between +auxin and −auxin, where *P* < 0.05. (*G***)** 4-thio UTP (4sUTP) nuclear run-on (NRO) analysis of UCPA and 3′ flanking (+1.8 kb) RNA from *MYC* in cells depleted of Xrn2-AID (1 h of auxin treatment)or not . Quantitation is shown for +auxin samples expressed relative to −auxin after normalizing to the level of unspliced *MYC* RNA. The asterisk denotes the difference between +auxin and −auxin, where *P* < 0.05. All error bars show standard deviation from at least three independent experiments.

Next, we tested the effect of Xrn2 loss on PAS cleavage and the stability of 3′ flanking region RNA from *MYC* and *ACTB* using quantitative RT–PCR (qRT–PCR). RNA was isolated over the same time course as for the Western blot in Figure 1E, and primers were used to detect non-PAS-cleaved (UCPA) RNA or 3′ flanking transcripts (Fig. 2A). An accumulation of 3′ flanking region RNA was seen for both genes by 30 min of auxin treatment. An even greater effect was seen after 60 min that was maintained (but not enhanced) after 120 min. In contrast, Xrn2-AID loss had no obvious effect on PAS cleavage, as no accumulation of UCPA species was observed for either gene at any time point. This experiment shows that in these two cases, Xrn2 degrades RNA beyond the PAS without affecting PAS cleavage. The latter conclusion is further supported by observations that Xrn2-AID loss has no impact on the recruitment of the polyadenylation factor Pcf11 to *ACTB* (Supplemental Fig. 2A). Importantly, 3′ flanking region RNA was stabilized only in the combined presence of the AID tag, Tir1, and auxin, showing that no individual factor indirectly causes the effect (Supplemental Fig. 2B). These findings are unlikely to result from secondary effects due to the speed of Xrn2-AID depletion, especially by comparison with RNAi, with the near-complete elimination of Xrn2-AID revealing function without overexpression of the inactive protein.

We then sought to test the generality of the effects seen on Xrn2-AID loss using nuclear RNA sequencing (RNA-seq) carried out on *XRN2-AID* cells treated with auxin or untreated. We also performed this analysis on a HCT116 cell line that was unmodified at *XRN2* and grown in the absence of auxin. Analysis of individual gene tracks confirmed the effect on *MYC* and *ACTB,* where an enhanced signal beyond their PASs was observed upon Xrn2-AID elimination (Fig. 2B). Further examples of Xrn2 effects are shown for *E2F6* and *RPL30* (Fig. 2C). *XRN2-AID* cells grown in the absence of auxin gave slightly elevated levels of 3′ flanking RNA as compared with cells unmodified at *XRN2*, suggesting that Xrn2-AID can carry out almost all 3′ flanking RNA degradation. Interestingly, strong effects of Xrn2 depletion were seen downstream from where Drosha cleaves microRNA (miRNA) precursors (Supplemental Fig. S3A,B), showing other ways of Xrn2 substrate generation.

Metagene plots were then generated for protein-coding genes that were separated from any reads within 3 kb of their transcription start site (TSS) and 7 kb of the PAS (denoted as TES [transcript end site]). This left 4701 genes for analysis and revealed a clear enhancement of 3′ flanking region RNA upon auxin treatment of *XRN2-AID* cells (Fig. 2D). Xrn2-AID samples obtained in the absence of auxin showed slightly raised levels of 3′ flanking region RNA compared with the cell line unmodified at *XRN2*, arguing that reduced levels of Xrn2-AID do not cause significant readthrough defects. Metagene plots generated from an independent biological replicate showed a similar result (Supplemental Fig. 3C). We note that Xrn2-AID loss is associated with a slight reduction in reads upstream of the PAS, potentially reflecting mildly reduced gene expression that might be caused by Pol II recycling defects. Finally, closer analysis of the TES (PAS) region showed that read counts at this position are similar in all samples (Fig. 2E; Supplemental Fig. 3D). This again suggests that major PAS cleavage defects are not widespread following Xrn2 loss, which is consistent with the analysis of *MYC* and *ACTB* shown above.

### Xrn2 degrades 3′ flanking RNA cotranscriptionally and promotes termination

The validity of the torpedo model of termination depends on cotranscriptional degradation of 3′ flanking region RNA taking place (Connelly and Manley 1988; Proudfoot 1989), but this has not been shown for Xrn2. To address this, we immunoprecipitated Pol II-associated RNA following cross-linking of *XRN2-AID* cells treated with auxin or untreated and analyzed it by qRT–PCR (Fig. 2F). Levels of UCPA RNA and 3′ flanking region RNA produced from *MYC* were assayed, and Xrn2 loss caused a substantial increase in the latter but not the former. This is consistent with Xrn2 involvement in the cotranscriptional degradation of 3′ flanking region RNA.

As a second measure of cotranscriptional degradation, we isolated nuclei from control or auxin-treated *XRN2-AID* cells and subjected them to nuclear run-on (NRO) analysis in the presence of 4-thio UTP (4sUTP). In this experiment, transcriptionally engaged Pol II was allowed to run on and label the 3′ ends of nascent transcripts in vitro. These were purified via linkage of biotin onto 4sUTP followed by streptavidin capture (see the Materials and Methods) and subjected to qRT–PCR to analyze UCPA and 3′ flanking region transcripts from *MYC* (Fig. 2G). This experiment yielded a result similar to that shown in Figure 2F in that Xrn2 loss increased 3′ flanking region RNA but not UCPA transcripts. The analysis of additional genes confirmed the role of Xrn2 in cotranscriptional degradation of 3′ flanking region RNA (Supplemental Figure 3E,F). Finally, stable integration of wild-type or catalytically inactive (D235A) *XRN2* into *XRN2-AID* cells demonstrated that both RNA degradation and termination defects caused by Xrn2-AID elimination are completely rescued by wild-type Xrn2 but not by D235A (Supplemental Fig. 4). The Xrn2 effects on transcriptional termination therefore require its exoribonuclease function.

### Mammalian native elongating transcript sequencing (mNET-seq) reveals a global termination defect on Xrn2 loss

Next, we precisely interrogated the global function of Xrn2 in transcriptional termination using mNET-seq (Nojima et al. 2015). In this method, the position of Pol II is revealed genome-wide at single-nucleotide resolution through its immunoprecipitation and the deep sequencing of RNA extracted from its active site. An antibody was used to capture all forms of Pol II.

**Figure 3.**
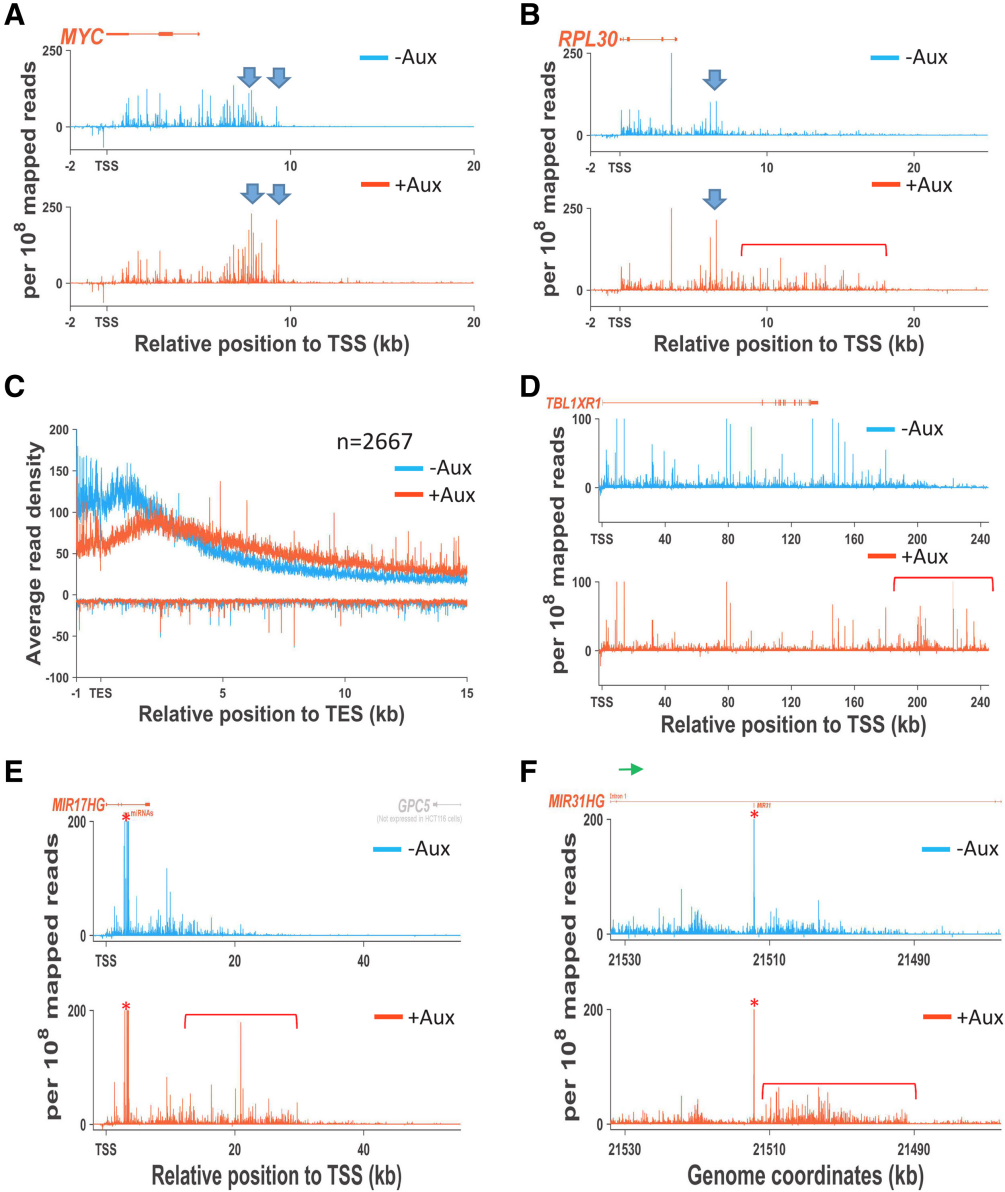
(*A*) *MYC* mNET-seq trace from *XRN2-AID* cells treated (orange) with auxin or untreated (blue) for 2 h. The *X*-axis shows a position relative to the gene TSS in kilobases. Reads are plotted as abundance per 10^8^ reads. Blue arrows denote a signal enhanced in the absence of Xrn2. (*B*) As in *A* but for *RPL30*. Additionally, the red bracket marks readthrough upon Xrn2 loss. (*C*) Metagene plot to analyze transcriptional termination on protein-coding genes in mNET-seq data from *XRN2-AID* cells grown with or without auxin. The average read density is shown over positions extending from 1 kb upstream of the TES to 15 kb downstream. The signal less than zero is transcription from the opposite strand, which is at or close to background. (*D*) As in *A* but for *TBL1XR1*. The red bracket denotes the region of extended readthrough. (*E*) As in *A* but for *MIR17HG*. In this case, a red asterisk marks the miRNA cleavage events, and a red bracket marks readthrough. (*F*) As in *E* but for *MIR31HG*. In each diagram, the expressed gene is shown in orange, with nonexpressed genes in gray.

*MYC* and *RPL30* mNET-seq profiles are shown in Figure 3, A and B (*ACTB* in Supplemental Fig. 5A). In cells not treated with auxin, termination occurs downstream from the PAS, where the mNET-seq signal reaches background. When Xrn2 is eliminated, a clear termination defect is observed, and, due to the high resolution of mNET-seq, it is possible to visualize two manifestations of this. First, where flanking region signal is detected in control cells, it is frequently elevated over the same positions in cells lacking Xrn2. This can be seen in the *MYC* and *RPL30* examples in Figure 3, A and B (blue arrows), and is consistent with polymerase stalling over termination regions facilitating termination by Xrn2. While this provides evidence that Xrn2 might not always have to pursue a still-transcribing Pol II, an additional effect of Xrn2 loss is an enhanced mNET-seq signal beyond where termination takes place in control cells. An example of this is marked by the red bracket on the *RPL30* gene plot in Figure 3B and suggests that normal termination sites can be ignored, with polymerases potentially having escaped pursuit by Xrn2.

We next addressed the generality of Xrn2 function in termination by generating metagene plots from control and auxin-treated cells. We analyzed expressed genes separated from upstream and downstream reads by at least 1 and 15 kb, respectively, which revealed a general transcriptional termination defect upon loss of Xrn2 (Fig. 3C). Interestingly, mNET-seq signal declined even in the absence of Xrn2, suggesting the existence of termination mechanisms that do not depend on it. The metagene plot of a separate biological replicate of this experiment showed the same general termination defect upon Xrn2-AID loss (Supplemental Fig. 5B). Interestingly, some genes were especially sensitive to Xrn2 elimination and showed more extensive readthrough than the genome-wide trend—as exemplified by *TBL1XR1* in Figure 3D. Nuclear RNA-seq analysis confirmed the extended readthrough over *TBL1XR1* (Supplemental Fig. 5C).

PAS cleavage is not the only mechanism to generate RNA 3′ ends. For instance, Drosha processes miRNAs, and a small number of noncoding RNA genes use this mechanism of 3′ end formation (Dhir et al. 2015). We tested whether Xrn2 promoted termination of two examples of these long noncoding primary miRNAs (lnc-pri-miRNAs): *MIR17HG* and *MIR31HG* (Fig. 3E,F). Cotranscriptional miRNA cleavage is visible (Fig. 3E,F, red asterisks) in both cases due to the known capacity of mNET-seq to detect Drosha cleavage products that remain associated with transcribing Pol II (Nojima et al. 2015). For *MIR17HG*, nascent transcription is detected in Xrn2 depleted samples beyond where termination occurs in the control experiment. There is also a higher read density beyond the *MIR31HG* miRNA sequence upon Xrn2 loss, with a noticeable defect emphasized by the reduced read count upstream of the Drosha cleavage site. This supports the notion that Xrn2 promotes efficient transcriptional termination from multiple cleavage processes, as suggested previously (Fong et al. 2015).

### Transcriptional termination on Histone and small nuclear RNA (snRNA) genes is unaffected by Xrn2 loss

**Figure 4.**
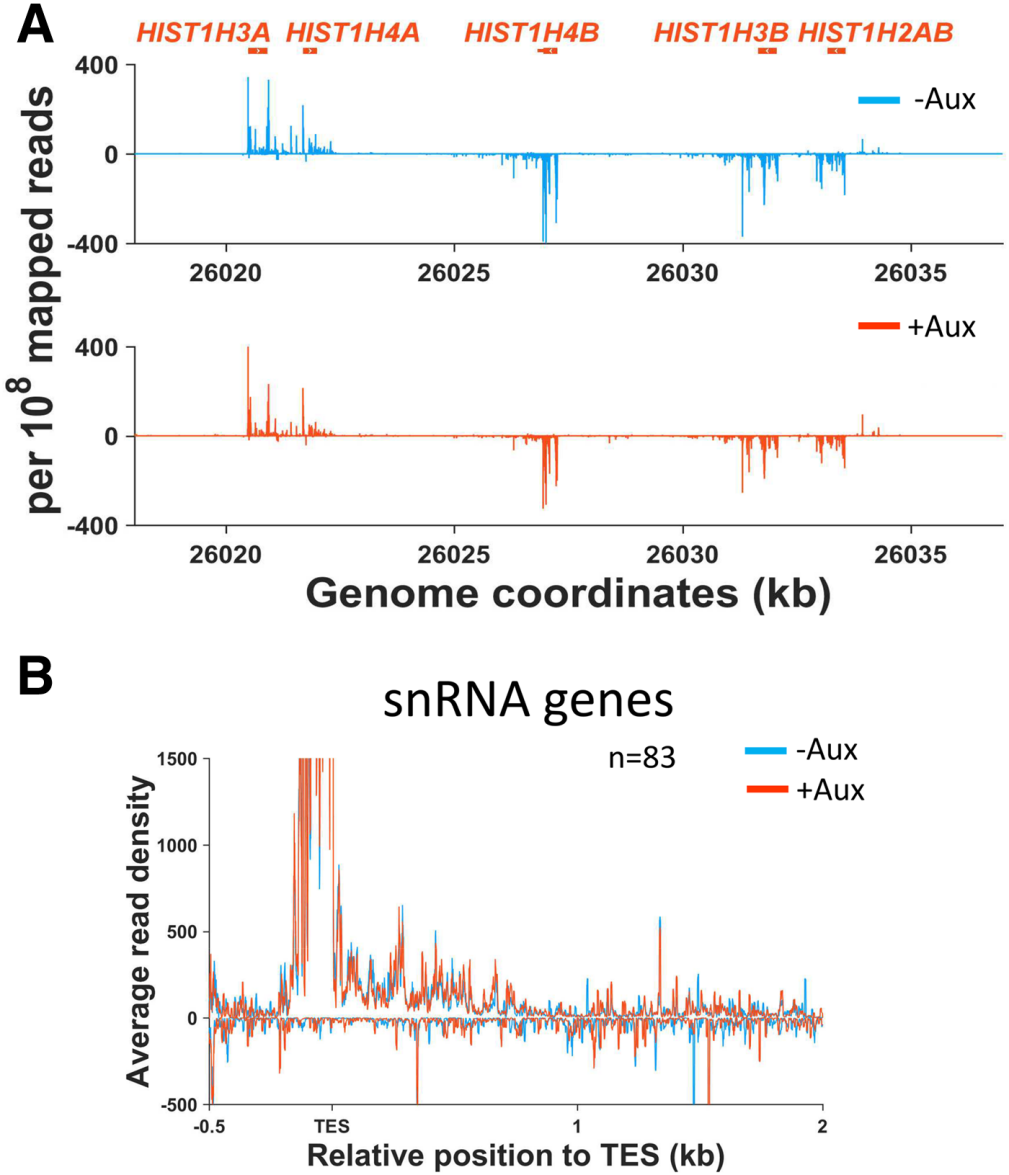
(*A*) mNET-seq profiles over the *HIST1* cluster from *XRN2-AID* cells treated with auxin or untreated. The *Y*-axes show signals per 10^8^ mapped reads. It should be noted that reads <0 represent examples of Histone genes expressed on the opposite strand. (*B*) mNET-seq metagene analyses of snRNA genes from *XRN2-AID* cells treated with auxin or untreated. The *Y*-axes show average read density and are scaled to zoom into the termination region where signals are much lower than the snRNA gene body.

Although not polyadenylated, Histone RNAs also use CPSF for 3′ end formation, which could provide an entry site for Xrn2 (Dominski et al. 2005; Kolev et al. 2008), and we were interested in whether this was the case. Figure 4A shows mNET-seq traces of the *HIST1* cluster in *XRN2-AID* cells treated with auxin or untreated. Interestingly, there is no impact of Xrn2 loss on transcriptional termination of any of the genes in this cluster, strongly suggesting that Xrn2 does not play a prominent role in Histone gene termination. This result was confirmed for other examples of Histone genes, and, similarly, RNA-seq showed little to no effect of Xrn2 elimination on 3′ flanking region RNA deriving from these genes (Supplemental Fig. 6). snRNAs also undergo 3′ end cleavage by the integrator complex, and this may also precede Xrn2 activity (Baillat et al. 2005). However, as for Histone genes, our mNET-seq and RNA-seq analyses showed no major role for Xrn2 in their transcriptional termination or in the degradation of their 3′ flanking region transcripts (Fig. 4B; Supplemental Fig. 7). As such, 3′ end cleavage is not always sufficient to promote an Xrn2-dependent termination process.

### Conditional depletion of CPSF73 causes a strong PAS cleavage and termination defect

**Figure 5.**
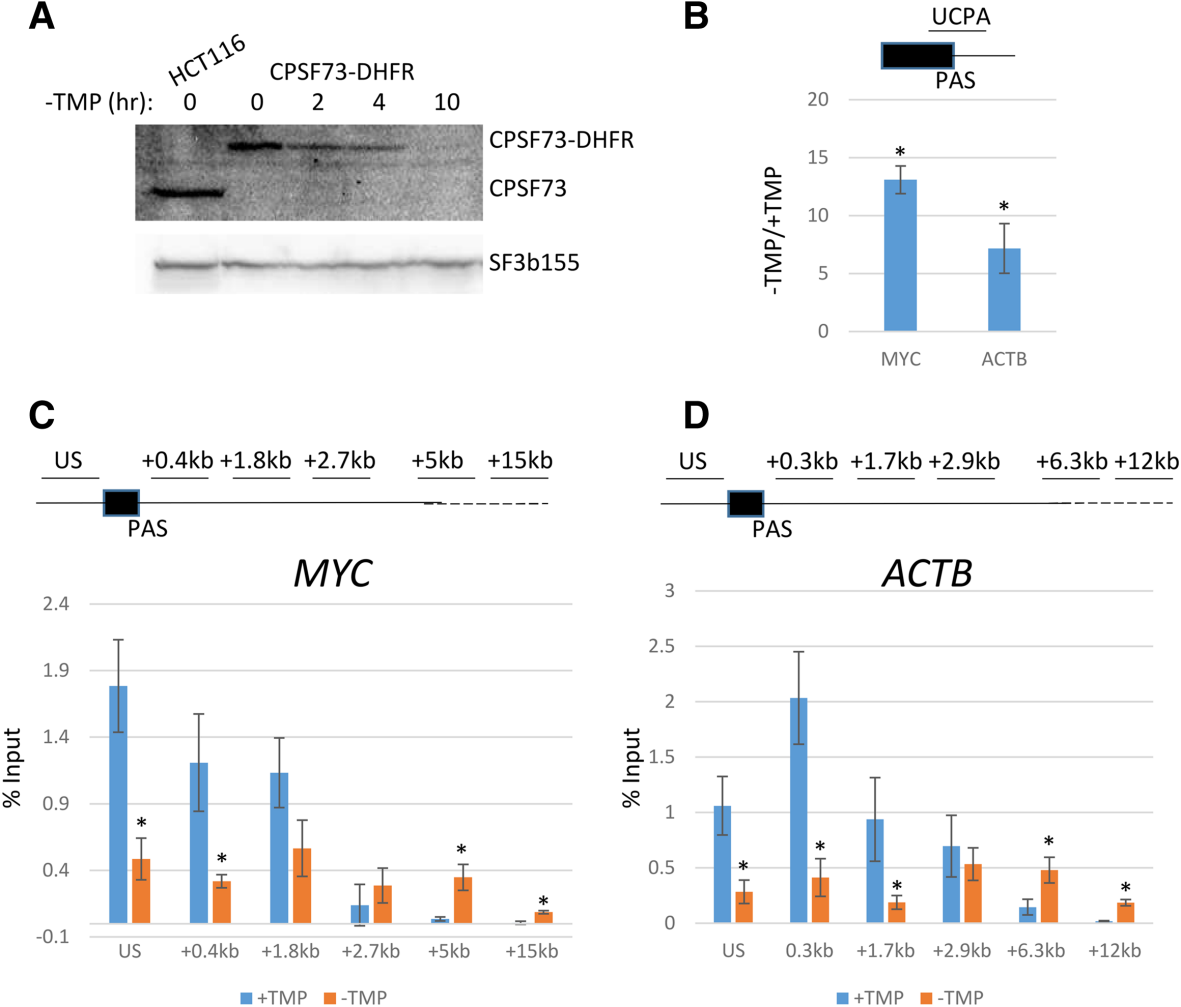
(*A*) Western blot showing successful tagging of *CPSF73* with DHFR and a time course of CPSF73-DHFR depletion in the absence of TMP. The *top* panel shows native CPSF73 in unmodified HCT116 cells and the higher-molecular-weight CPSF73-DHFR in CRISPR-modified cells. CPSF73-DHFR levels are depleted in the absence of TMP. SF3b155 was detected as a loading control. (*B*) qRT–PCR analysis of UCPA RNA from *MYC* or *ACTB* genes in CPSF73-DHFR cells grown in the presence or absence of TMP. Values are expressed relative to those obtained in cells grown in TMP after normalizing to unspliced RNA levels from each gene to account for any effects of transcription. Asterisks denote *P* < 0.05 for differences between +TMP and −TMP. (*C*) Pol II chromatin immunoprecipitation (ChIP) on *MYC* in *CPSF73-DHFR* cells grown in the presence or absence of TMP. Values are expressed as the percentage of input, and asterisks denote differences between +TMP and −TMP samples with *P* < 0.05. (*D*) As in *C* but on *ACTB*. All error bars show standard deviation from at least three independent experiments.

For Xrn2 to function in termination, RNA cleavage is required, and this presumably occurs most often at the PAS. CPSF73 is the PAS endonuclease in humans, and its depletion by RNAi causes strong termination defects genome-wide, confirming its general function in the process (Nojima et al. 2015). However, depletion of CPSF73 cannot establish whether its catalytic center or physical presence underlies its function in termination. To begin testing this, we generated cells in which the PAS endonuclease CPSF73 could be manipulated in a manner similar to Xrn2-AID. As we were unable to make an AID-tagged version of CPSF73, we tagged its C terminus with an *Escherichia coli* DHFR-based degron using the system used for Xrn2-AID (Iwamoto et al. 2010; Sheridan and Bentley 2016). In this system, cells are grown in the presence of trimethoprim (TMP), the withdrawal of which triggers degradation of the tagged protein. Western blotting confirmed homozygous tagging of *CPSF73* with *DHFR*, as CPSF73-DHFR was seen to migrate at a higher molecular weight than the native protein for which there was no signal in the CRISPR-modified cell line (Fig. 5A). Withdrawal of TMP from the medium promoted near elimination of CPSF73-DHFR after 10 h. This rate of depletion is slower than for Xrn2-AID but more than sevenfold faster than what we used previously for functional depletion of CPSF73 by RNAi (Davidson et al. 2014).

We tested the impact of CPSF73-DHFR elimination on 3′ end processing of *MYC* and *ACTB* transcripts by qRT–PCR of total RNA from *CPSF73-DHFR* cells grown in the presence or absence of TMP (Fig. 5B). For both genes, there was a significant reduction of PAS cleavage, demonstrated by an accumulation of UCPA RNA. Notably, the magnitude of effect (sevenfold to 12-fold) was threefold to fourfold greater than we observed previously by RNAi of CPSF73 (Davidson et al. 2014), highlighting the enhanced effects gained from this system.

To analyze the effect of CPSF73 depletion on termination, Pol II chromatin immunoprecipitation (ChIP) was performed in *CPSF73-DHFR* cells grown in the presence or absence of TMP. Pol II occupancy was monitored downstream from *MYC* and *ACTB* (Fig. 5C,D). In both cases, CPSF73 loss caused a general reduction in transcription, as evidenced by the lower Pol II signal upstream of the PAS (denoted as US). This is consistent with observations that PAS mutations or polyadenylation factor depletion negatively impacts transcription (Mapendano et al. 2010). Despite this, a large termination defect was evident on both genes through the accumulation of Pol II beyond the normal site of termination.

### CPSF73 elimination causes more extensive readthrough than loss of Xrn2

**Figure 6.**
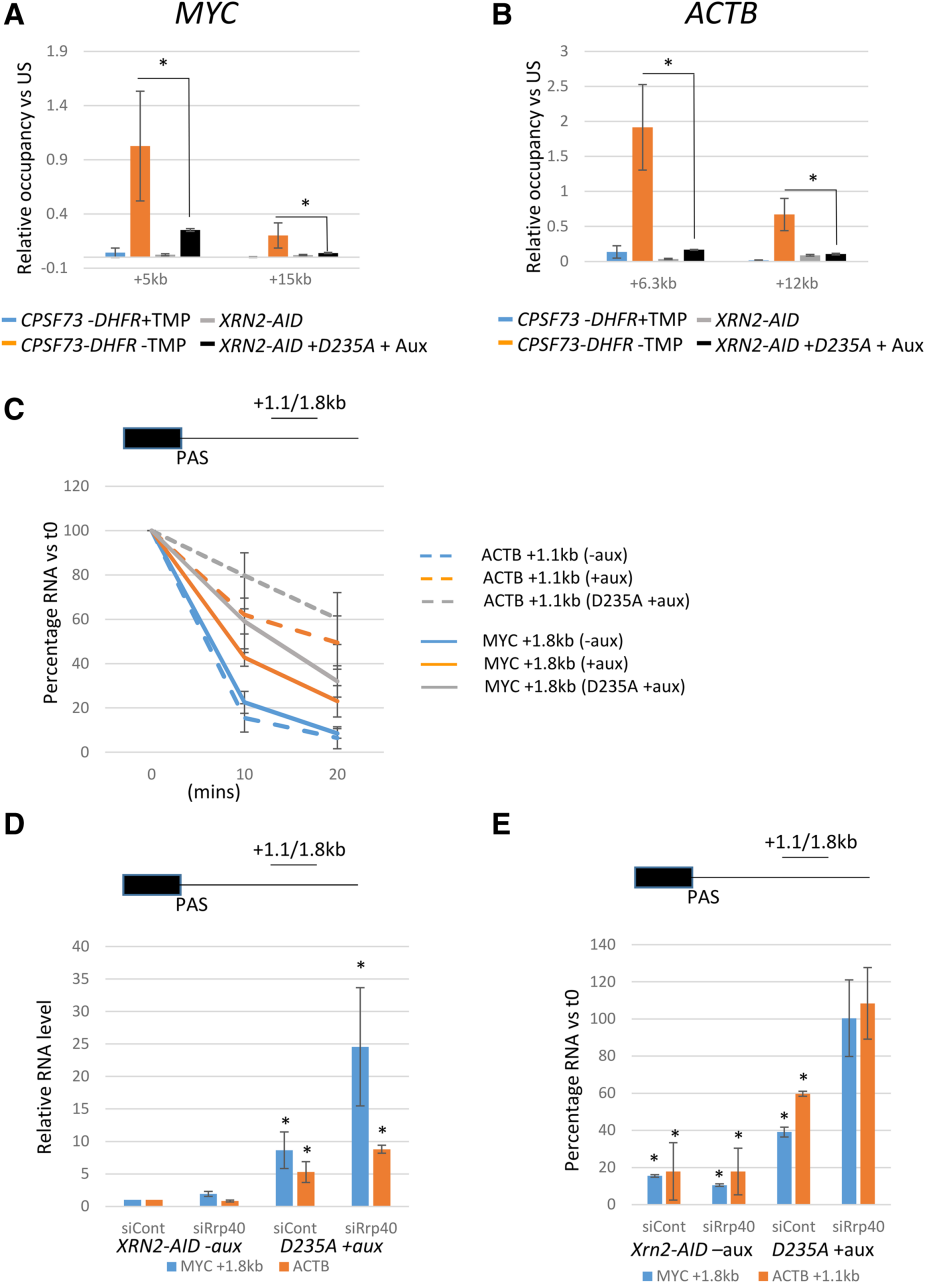
(*A*) Analysis of Pol II occupancy at +5 and +15 kb beyond the *MYC* PAS expressed relative to that upstream of the PAS (US) in *CPSF73-DHFR* cells ±TMP, *XRN2-AID* cells, and *XRN2-AID* + *D235A* cells +auxin (1 h). Asterisks denote *P* < 0.05 between CPSF73-DHFR – TMP and *XRN2-AID* + *D235A* + auxin. (*B*) As in *A* but for 6.3 and 12 kb beyond the *ACTB* PAS. (*C*) qRT–PCR analysis of *ACTB* and *MYC* 3′ flanking region RNA degradation in *XRN2-AID* cells treated with auxin or *D235A* cells treated with auxin (all auxin for 1 h) followed by 10 or 20 min of actinomycin D (Act D) treatment. For each sample set, RNA levels are expressed relative to that recovered at t0. (*D*) qRT–PCR analysis of *ACTB* and *MYC* 3′ flanking region RNA in control or human Rrp40 (hRrp40) siRNA-treated *XRN2-AID* cells or *D235A* cells treated with auxin (all auxin for 1 h). RNA levels are expressed as a fold change relative to those recovered in *XRN2-AID* cells treated with control siRNA following normalization to the level of unspliced *MYC* or *ACTB* transcripts. Asterisks denote *P* < 0.05 versus *XRN2-AID* cells treated with control siRNA. (*E*) qRT–PCR analysis of *ACTB* and *MYC* 3′ flanking region RNA under the conditions used in *D* but after 20 min of Act D treatment. Values are expressed as a percentage of RNA remaining under each condition relative to the amounts recovered in each sample at t0. Asterisks denote *P* < 0.05 versus the 0 time point. All error bars show standard deviation from at least three independent experiments.

We next tested whether CPSF73 and Xrn2 produced differential effects on readthrough transcription. For this, Pol II ChIP was compared for *CPSF73-DHFR* cells ±TMP, on *XRN2-AID* cells, and on *D235A XRN2-AID* cells +auxin (Fig. 6A,B). *D235A XRN2-AID* cells stably express catalytically inactive Xrn2 that is not sensitive to auxin. When these cells are treated with auxin, 5′ → 3′ degradation of readthrough RNA and termination are more strongly impaired than in auxin-treated *XRN2-AID* cells (Supplemental Fig. 4). Pol II occupancy over extended readthrough regions of *MYC* and *ACTB* was plotted relative to the signal from upstream of the PAS. For both genes, CPSF73 depletion resulted in greater signals over extended positions than elimination of Xrn2 function, suggesting that termination is more adversely effected by loss of CPSF73. qRT–PCR analysis of readthrough RNA over the same positions confirmed this result (Supplemental Fig. 8A). Inhibition of CPSF30 function by influenza NS1A protein (Nemeroff et al. 1998) also caused more extensive transcriptional readthrough than Xrn2, further arguing for a more crucial function of CPSF in promoting termination (Supplemental Fig. 8B,C).

Although auxin-treated *D235A* cells represent the scenario most lacking in 5′ → 3′ degradation of RNA, the smaller effect on termination relative to CPSF73 loss may be due to incomplete Xrn2 depletion or other 5′ → 3′ nucleases acting in its absence. To address this, we analyzed the turnover rate of 3′ flanking region transcripts from *MYC* and *ACTB* in more detail. A time course was used in *XRN2-AID* cells treated with auxin or untreated and in *D235A* cells treated with auxin following transcriptional inhibition by actinomycin D (Act D) (Fig. 6C). In *XRN2-AID* cells not treated with auxin, Act D induced a strong reduction in the level of 3′ flanking region RNA, consistent with rapid degradation. The addition of auxin resulted in greater recovery of 3′ flanking region RNA following Act D treatment that was more pronounced in *D235A* cells treated with auxin. This confirms the role of Xrn2 in their degradation. However, degradation was incompletely blocked by Xrn2 elimination, as ∼40%–60% of these transcripts were still degraded after transcriptional inhibition even in auxin-treated *D235A* cells.

The degradation of 3′ flanking region RNA in auxin-treated *D235A* cells could be by alternative 5′ → 3′ exonucleases or from the 3′ end by the exosome. To address this, we treated *D235A* cells with control or human Rrp40 (hRrp40)-specific siRNAs before auxin addition (Fig. 6D; Supplemental Fig. 9A,B). The same experiment was performed on *XRN2-AID* cells not treated with auxin to determine any exclusive effects of hRrp40 depletion. We first tested the effects of these conditions on the levels of *MYC* and *ACTB* 3′ flanking region RNA. hRrp40 depletion alone gave no substantial effect, whereas auxin treatment of *D235A* cells gave the expected strong accumulation. When hRrp40 was depleted from *D235A* cells treated with auxin, there was an accumulation of 3′ flanking region RNA above what was seen upon manipulation of Xrn2 that was most marked for *MYC* transcripts. Therefore, the exosome contributes to readthrough RNA degradation in the absence of Xrn2 function. The level of UCPA transcripts was similar under each of these conditions, arguing that PAS cleavage is unaffected (Supplemental Fig. 9C).

Next, the impact of the exosome on degradation of 3′ flanking RNAs was assessed after 20 min of Act D treatment (Fig. 6E). In the *XRN2-AID* sample treated with control siRNA, Act D treatment caused depletion of *ACTB* and *MYC* flanking transcripts as expected, and hRrp40 depletion gave a similar result. In auxin-treated *D235A* cells, ∼40%–60% of 3′ flanking region RNA was again degraded in the absence of Xrn2 function. Importantly, hRrp40 depletion from *D235A* cells grown in auxin essentially blocked degradation, as the level of RNA recovered after transcription inhibition was similar to before Act D addition. This shows that the exosome rather than other 5′ → 3′ exonucleases is responsible for the degradation of RNA that occurs in the absence of functional Xrn2. As such, auxin treatment of D235A cells effectively blocks degradation of 3′ flanking region transcripts from their 5′ ends. A similar result was obtained when transcription was inhibited using flavopiridol (Supplemental Fig. 9D). Act D time course analysis also revealed that CPSF73 elimination prevented turnover of 3′ flanking region RNA, arguing that PAS cleavage is necessary to promote their degradation (Supplemental Fig. 9E). These data argue that the differential effect of Xrn2 and CPSF73 on transcriptional termination is unlikely to be due to an incomplete block of 5′ → 3′ degradation when Xrn2 is manipulated. As such, they support the existence of additional termination mechanisms that occur in the absence of 5′ → 3′ degradation.

### A CPSF73 active site mutant cannot support efficient transcriptional termination

**Figure 7.**
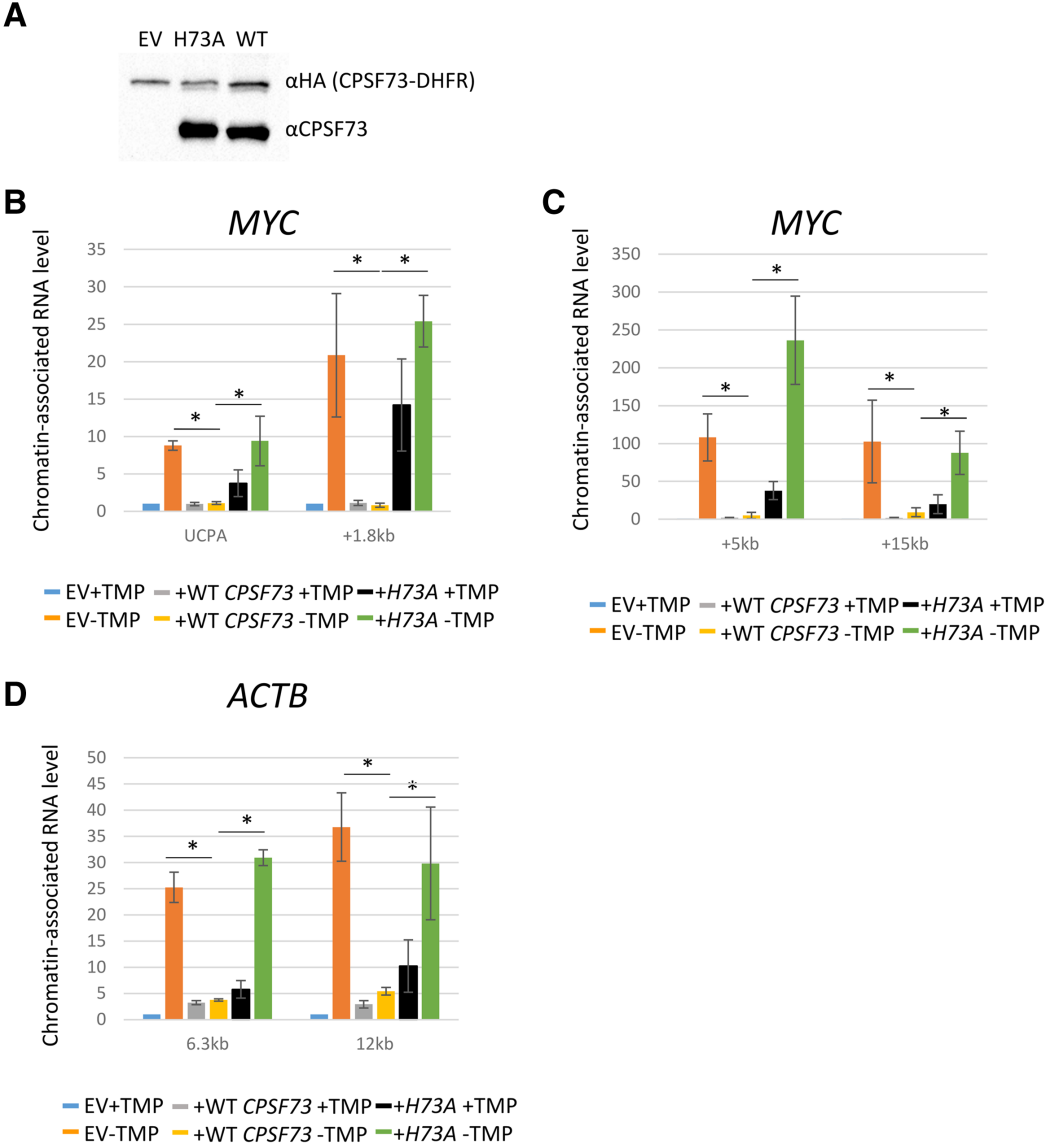
(*A*) Western blotting of *CPSF73-DHFR* cells transfected with H73A CPSF73, wild-type (WT) CPSF73, or empty vector (EV) and probed with anti-HA (to detect CPSF73-DHFR) or anti-CPSF73 (to additionally detect protein derived from transfected constructs). (*B*) qRT–PCR analysis of chromatin-associated RNA isolated from *CPSF-DHFR* cells transfected with empty vector, wild-type, or H73A DHFR ± TMP. Primers were used to detect UCPA Myc RNA or RNA from +1.8 kb beyond the PAS. Values are expressed relative to those in empty vector samples in the presence of TMP after normalizing to unspliced RNA levels. Asterisks display *P* < 0.05 for comparison of the ability or inability of wild-type or H73A CPSF73 to restore termination in relation to the situation lacking CPSF73-DHFR. (*C*) As in *B* but showing signals for +5 and +15 kb beyond the *MYC* PAS. (*D*) As in *B* but detecting RNA from positions +6.3 or +12 kb beyond the *ACTB* PAS. All error bars show standard deviation from at least three independent experiments.

A primary termination pathway involving Xrn2 predicts a requirement for PAS cleavage. To test whether active CPSF73 is required for termination, we generated plasmids containing either wild-type *CPSF73* or a point-mutated derivative (H73A) shown previously to have diminished nuclease activity (Kolev et al. 2008). The plasmid system was used because repeated attempts to stably integrate H73A into *CPSF73-DHFR* cells failed, potentially because of its deleterious effect. Plasmids also incorporated puromycin selection markers to enrich for transfected cells. Western blotting confirmed similar expression of wild-type and H73A proteins in *CPSF73-DHFR* cells and the expected absence of endogenous-sized CPSF73 in empty vector transfected samples (Fig. 7A).

To test the ability of H73A to function in termination, *CPSF73-DHFR* cells were transfected with empty vector, wild type, or H73A. Transfected cells were then enriched for by puromycin selection before removal (or not) of CPSF-DHFR via 10 h of TMP withdrawal. Chromatin-associated RNA was then isolated to study termination via the extent of nascent RNA transcription, which was assayed by qRT–PCR for *MYC* and *ACTB* genes (Fig. 7B–D). In empty vector transfected cells, TMP withdrawal induced the expected accumulation of UCPA RNA and a strong enhancement of readthrough transcripts extending beyond the PAS. These readthrough defects were substantially suppressed in the absence of TMP by wild-type CPSF73. However, H73A expression caused accumulation of readthrough RNA even in the presence of CPSF73-DHFR. This dominant-negative effect indicates that H73A successfully competes with CPSF73-DHFR from PAS cleavage complexes, causing impaired termination. This was confirmed by withdrawal of TMP that showed H73A to be incapable of restoring PAS cleavage or readthrough RNA levels to normal levels. These data strongly suggest that active CPSF73 is required for efficient termination.

## Discussion

Our study reveals a clear role for CPSF73 activity and 5′ → 3′ degradation in efficient termination on protein-coding genes as envisioned by the torpedo model. They are most consistent with a primary mechanism in which PAS site cleavage precedes cotranscriptional degradation of Pol II-associated RNA by Xrn2. However, we also observed some termination in situations where 5′ → 3′ degradation of RNA was blocked, arguing for alternative secondary mechanisms. In particular, ablation of CPSF73 activity caused more readthrough than seen on loss of Xrn2, suggesting additional roles for CPSF73 in termination. The observation that miRNA cleavage is capable of promoting Xrn2-dependent termination argues that RNA cleavage may more widely underpin the process beyond protein-coding genes.

Previous reports have reached different conclusions on the role of Xrn2 in termination. Originally, RNAi of Xrn2 caused a termination defect on transfected β-globin plasmids, while a subsequent global analysis found no genome-wide function for Xrn2 in termination at gene 3′ ends using mNET-seq (West et al. 2004; Nojima et al. 2015). An explanation for this came through observations that RNAi of Xrn2 caused termination defects when catalytically inactive Xrn2 was also expressed (Fong et al. 2015). Our results support the view that trace levels of active Xrn2 can provide false negative results in RNAi experiments because Xrn2-AID is virtually eliminated in our system, with its levels likely falling below a critical threshold. Moreover, although Xrn2-AID protein is at substantially reduced levels compared with native Xrn2, this is still sufficient to promote termination, suggesting that a fraction of normal levels supports this function. Finally, expression of inactive Xrn2 in *XRN2-AID* cells has a dominant-negative effect on termination in our system (Supplemental Fig. 4E). These observations may be of importance beyond Xrn2, as they suggest that a degron-based approach can yield a fuller repertoire of functions for some proteins than RNAi alone.

Another finding in our study is that termination is not readily observed in the absence of CPSF73 activity, suggesting that PAS cleavage is required. In vitro experiments suggest that PAS cleavage is not absolutely required for termination (Zhang et al. 2015). However, additional cellular factors may be absent from in vitro systems. Moreover, RNA degradation improved termination in that system, consistent with our finding on the importance of Xrn2 in cells. We do note that H73A CPSF73 has been shown to immunoprecipitate other CPSF components slightly less efficiently than wild-type CPSF73 (Kolev et al. 2008). This means that the presence of incomplete or unstable CPSF complexes might account for the inability of the H73A mutant to promote termination. If this is true, then it would identify CPSF assembly or activation as providing a crucial function in the process rather than PAS cleavage itself. This would still be an important observation, but we favor PAS cleavage as important for several reasons. First, H73A proved an effective dominant-negative inhibitor of PAS cleavage. Second, partial defects in complex formation might be expected to result in partial termination defects instead of the very strong effect caused by exclusive H73A expression. Moreover, recent results show that polyadenylation factors, exemplified by CstF64, assemble on inactive intronic PASs, but this is insufficient to cause termination unless cleavage is activated by U1 snRNA inhibition (Oh et al. 2017). Finally, the widespread requirement for Xrn2 in efficient termination is most readily explained by PAS cleavage preceding its action.

We also suggest that CPSF73 is required for termination even in the absence of Xrn2. The evidence for this conclusion is that the termination defect is larger upon loss of CPSF73 than when Xrn2 is absent. This could be due to allosteric effects induced by CPSF assembly or activity. Alternatively, such termination could be via the RNA:DNA helicase activity of Senataxin (Skourti-Stathaki et al. 2011), given that its budding yeast homolog, Sen1, can terminate polymerase in purified systems (Porrua and Libri 2013). The exosome may also terminate Pol II by degrading RNA that protrudes from the front of backtracked polymerase (Lemay et al. 2014). Our data argue that these possibilities, including an allosteric mechanism, would require PAS cleavage, given the inability of inactive CPSF73 to support termination. A termination mechanism underpinned by cleavage may also apply following miRNA cleavage. We show an Xrn2 effect on this process; however, the readthrough caused is less than previously observed when miRNA cleavage was prevented by Drosha depletion (Dhir et al. 2015). Drosha depletion caused *MIR17HG* transcription to enter the downstream *GPC5* gene, whereas transcription terminates before this point following Xrn2 loss (Fig. 3E).

While it is difficult to interrogate some molecular details of termination in cells, important principles are consolidated here. In particular, we provide strong evidence that PAS cleavage and cotranscriptional degradation of Pol II-associated RNA are key components of the most efficient termination mechanism. Our results align with predictions of the torpedo model made using highly purified in vitro systems, where it was shown that Xrn2-, Rat1-, and Xrn1-mediated RNA degradation terminates Pol II (Park et al. 2015). In those cases, termination improved when Pol II-associated RNA was longer or when Pol II progression was prevented by nucleotide misincorporation, suggesting that nuclease momentum or polymerase stalling may facilitate the process in cells. Polymerase backtracking over termination regions was inferred from transient transcriptome sequencing (TT-seq) (Schwalb et al. 2016). Moreover, our mNET-seq shows signal accumulation over termination regions that may result from pausing or backtracking. As this signal is often enhanced by loss of Xrn2 (denoted by the blue arrows in Fig. 3), polymerases prone at these sites may be more vulnerable to termination by Xrn2. As we also observed a signal beyond termination sites upon loss of Xrn2, it will be interesting to establish whether this represents polymerases that resume transcription following pausing or those normally terminated by a pause-independent process. In sum, our results provide important details on the termination mechanism in human cells, especially regarding CPSF73 and Xrn2 activities. Our AID system provides a rationale for why RNAi of Xrn2 led to controversy over its role in the process, and our DHFR approach gives strong evidence that PAS cleavage precedes termination.

## Materials and methods

### Plasmids, primers, and DNA sequences

Primer sequences used for ChIP and qRT–PCR, sequences of repair templates, homology arms, and guide RNA target sites are provided in the Supplemental Material.

### Antibodies

The antibodies used were Pol II (CMA601; MBL Technologies), CPSF73 (Abcam, ab72295), CPSF73 for Figure 5A (Bethyl Laboratories, A301-090A), Flag (Sigma, F3165), HA (Roche, 3F10), Xrn2 (Bethyl Laboratories, A301-101), SF3b155 (Abcam, ab39578), Myc (Sigma, 9E10), Pcf11 (Bethyl Laboratories, A303-705 and A303-706), and NS1A (gift from Aldolfo Garcia-Sastre).

### Cell culture

HCT116 cells were maintained in DMEM with 10% fetal calf serum. Transfections were with JetPrime (polyplus). For CRISPR, 1 µg of guide RNA plasmid and 1 µg of each repair plasmid were transfected into six-well dishes. Twenty-four hours later, culture medium was changed, and, a further 24 h later, cells were split into a 100-mm dish containing 800 µg/mL neomycin and 150 µg/mL hygromycin. After ∼10 d of selection, single colonies were transferred to a 24-well plate and screened by PCR or Western blotting. The presence of repair cassettes at *XRN2* or *CPSF73* was confirmed by Sanger sequencing. An optimized sleeping beauty transposon system (Kowarz et al. 2015) was used to generate Tir1-expressing parental cell lines and cells in which Xrn2 derivatives were stably transfected. A 24-well dish was transfected with 300 ng of sleeping beauty plasmid (derived from pSBbi-puro/pSBbi-blast)) and 100 ng of pCMV(CAT)T7-SB100. Twenty-four hours later, cells were put under selection with 1 µg/mL puromycin or 20 µg/mL blasticidin. For Tir1-expressing cells, single colonies were isolated; for Xrn2 rescue experiments, the entire population was studied. Auxin (Sigma) was added to 500 nM for 60 min unless stated otherwise. TMP (Sigma) was maintained at 20 µM, and, for depletion, cells were grown in medium lacking TMP for 10 h unless stated otherwise. Act D and flavopiridol were used at 5 μg/mL and 1 μM, respectively.

### qRT–PCR

Tri reagent (Sigma) was used to isolate total RNA following the manufacturers’ guidelines, and RNA was treated with Turbo DNase (Life Technologies) for 1 h. In all cases, reverse transcription of 1 µg of RNA was primed with random hexamers using Protoscript II (New England Biolabs). qPCR was performed using Brilliant III (Agilent Technologies) in a Qiagen Rotorgene instrument. Comparative quantitation was used to establish fold effects.

### ChIP and RNA immunoprecipitation

For ChIP, one 100-mm dish of cells was cross-linked for 10 min in 0.5% formaldehyde, and cross-links were quenched in 125 mM glycine for 5 min. Cells were collected (500*g* for 5 min) and resuspended in 400 µL of RIPA buffer (150 mM NaCl, 1% NP40, 0.5% sodium deoxycholate, 0.1% SDS, 50 mM Tris-HCl at pH 8, 5 mM EDTA at pH 8). Samples were sonicated in a Bioruptor sonicator (30 sec on and 30 sec off) 10 times on high setting. Tubes were spun at 13,000 rpm for 10 min. Supernatant was then split into two and added to 30 µL of Dynabeads (Life Technologies) that had been incubated for 2 h with 3 µg of antibody or, as a control, mock-treated. Ten percent of the supernatant was kept for input. Immunoprecipitation was for 2–14 h at 4°C, and beads were then washed twice in RIPA, three times in high-salt wash buffer (500 mM NaCl, 1% NP40, 1% sodium deoxycholate, 100 mM Tris-HCl at pH 8.5), and once in RIPA. Samples were eluted (0.1 M NaHCO_3_ + 1% SDS), and cross-links were reversed overnight at 65°C. DNA was purified by phenol chloroform extraction and ethanol precipitation. Samples were generally resuspended in 100 µL of water, with 1 µL used per PCR reaction. For RNA immunoprecipitation, cross-links were reversed for 45 min at 65°C. RNA was purified by phenol chloroform extraction and ethanol precipitation followed by DNase treatment and reverse transcription.

### Chromatin RNA isolation

Nuclei were isolated from cells by resuspending cell pellets from a 100-mm dish in hypotonic lysis buffer (HLB; 10 mM Tris at pH 7.5, 10 mM NaCl, 2.5 mM MgCl_2_, 0.5% NP40). This was underlayered with HLB + 10% sucrose and spun at 500*g* for 5 min. Nuclei were resuspended in 100 μL of NUN1 (20 mM Tris-HCl at pH 7.9, 75 mM NaCl, 0.5 mM EDTA, 50% glycerol, 0.85 mM DTT). One milliliter of NUN2 (20 mM HEPES at pH 7.6, 1 mM DTT, 7.5 mM MgCl_2_, 0.2 mM EDTA. 0.3 M NaCl, 1 M urea, 1% NP40) was added before incubation for 15 min on ice with regular vortexing. Chromatin pellets were isolated by centrifugation at 13,000 rpm in a benchtop centrifuge, and RNA was isolated using Trizol.

### 4sUTP NRO

Nuclei were isolated as for chromatin-associated RNA. These were resuspended in an equal volume of 2× transcription buffer (40 mM Tris-HCl at pH 7.9, 300 mM KCl, 10 mM MgCl_2_, 40% glycerol). This was supplemented with rA, C, and G together with 4sUTP (final concentration ∼0.1 mM). Following incubation for 15 min at 30°C, RNA was isolated, and biotin linkage and capture were performed as described in Duffy et al. (2015) with some modification. RNA (15–20 µg) was biotinylated in a volume of 250 μL containing 10 mM HEPES (pH 7.5) and 5 µg of MTSEA Biotin-XX (Iris Biotech) dissolved in dimethyl formamide. After incubation in the dark for 90 min, biotinylated RNA was phenol chloroform-extracted and ethanol-precipitated. This was resuspended in RPB (300 mM NaCl, 10 mM Tris at pH 7.5, 5 mM EDTA) and incubated with 150 μL of streptavidin-coated paramagnetic particles (Promega) for 15 min. Beads were washed five times in 100 mM Tris-HCl (pH 7.4), 10 mM EDTA, 1 M NaCl, and 0.1% Tween-20 preheated to 60°C. RNA was eluted in 100 μL of 0.1 M DTT for 15 min at 37°C before final phenol chloroform extraction and ethanol precipitation.

### Nuclear RNA-seq

Following 1 h of auxin or mock treatment, nuclei were isolated as for chromatin-associated RNA. Nuclear RNA was extracted using Trizol reagent. rRNA was removed using Ribo-Zero Gold rRNA removal kit (Illumina) according to the user manual. Libraries were prepared using TruSeq stranded total RNA library preparation kit (Illumina) according to the manual and purified using Ampure XP beads (Beckman Coulter). Libraries were screened for fragment size and concentration by Tapestation D1000 (Agilent) and sequenced using HiSeq 2500 (Illumina).

Raw single-end 50-base-pair (bp) reads were screened for sequencing quality using FASTQC, adapter sequences were removed using Trim Galore (wrapper for Cutadapt), and trimmed reads <20 bp were discarded. Reads were aligned to the GRCh38 human genome using Hisat2 (Kim et al. 2015) with splice site annotation from Ensembl. Unmapped and low MAPQ reads were discarded. For metagene analyses, expression levels were calculated for each gene, and genes with low or no expression were removed. A transcriptional window was then applied (TSS − 3 kb and TES + 7 kb). Genes with overlapping reads in this window were discarded (Quinlan and Hall 2010). Metagene profiles were generated using the deeptools suite (Ramirez et al. 2016), with further graphical processing performed in the R environment (http://www.R-project.org). Normalized gene coverage plots were visualized using the Integrated Genome Viewer suite (Robinson et al. 2011).

### mNET-seq

A detailed description of the mNET-seq protocol can be found in the study by Nojima et al. (2016). *XRN2-AID* cells were treated for 2 h with auxin or left untreated. NEBNext small RNA libraries were sequenced using HiSeq 2500 (Illumina). Raw 50-bp paired-end sequences had adapter sequences removed using Trim Galore, and resultant reads with a quality of <20 and fragment size of <19 bp were discarded. Reads were aligned using HiSat2 against GRCh38 (Ensemble) with known splice site annotation (Gencode), and concordantly mapped read pairs were selected (Kim et al. 2015).

The mNET-seq traces used single-nucleotide resolution BAM files corresponding to the 3′ end of the RNA fragment (Nojima et al. 2015). For metagene profiles, gene expression was determined by converting raw read counts into transcripts per million (TPM) for each annotated gene (Li and Dewey 2011; Wagner et al. 2012; Liao et al. 2014). For protein-coding metaplots, genes were selected where no other expressed annotated gene overlapped the exclusion range (TES − 1250 bp to TES + 15,250 bp). For each nucleotide across the region, fragments were counted in a 5-bp sliding window and converted to TPM. The normalized metagene profiles represent the average nascent RNA fragment density against relative position from the TES. mNET-seq and RNA-seq data have been deposited with Gene Expression Omnibus (accession no. GSE109003).

## Supplementary Material

Supplemental Material
