# Supplementary material for: Xrn2 accelerates termination by RNA polymerase II, which is underpinned by CPSF73 activity
Source: Genes Dev. 2018 Jan 15;32(2):127–39. doi: 10.1101/gad.308528.117 (PMC5830926; doi:10.1101/gad.308528.117)
Supplement: Supplemental Material [file supp_32_2_127__index.html]

Xrn2 accelerates termination by RNA polymerase II, which is underpinned by CPSF73 activity — Supplemental Material 

# Xrn2 accelerates termination by RNA polymerase II, which is underpinned by CPSF73 activity

## Supplemental Material

- Supplemental\_material.pdf
