# Supplementary material for: Xrn2 accelerates termination by RNA polymerase II, which is underpinned by CPSF73 activity
Source: Genes Dev. 2018 Jan 15;32(2):127–39. doi: 10.1101/gad.308528.117 (PMC5830926; doi:10.1101/gad.308528.117)
Supplement: Supplemental Material [file supp_gad.308528.117_Supplemental_material.pdf]

Eaton_Supplementary Fig1

A

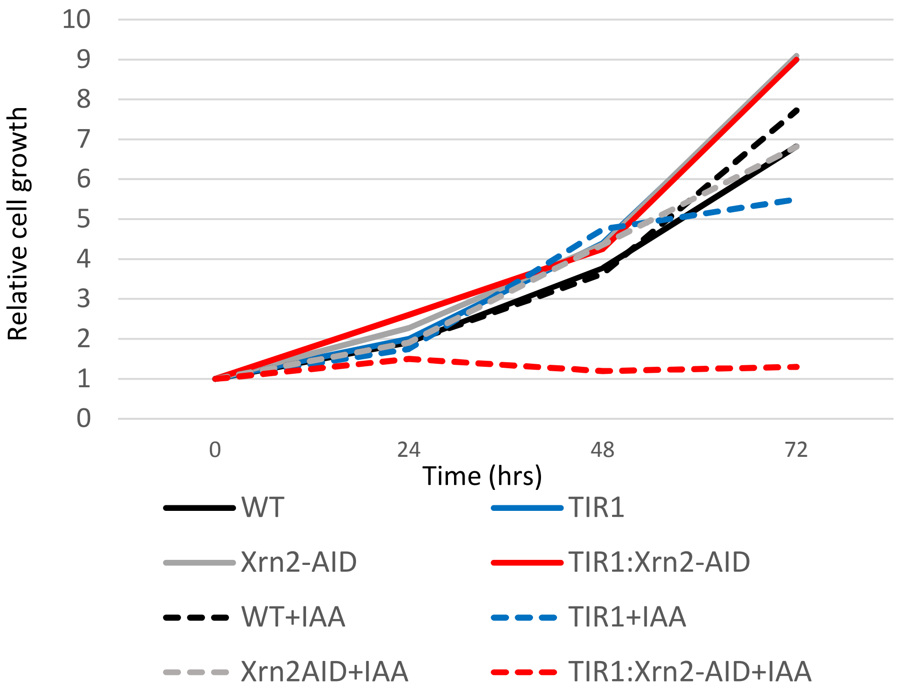

B

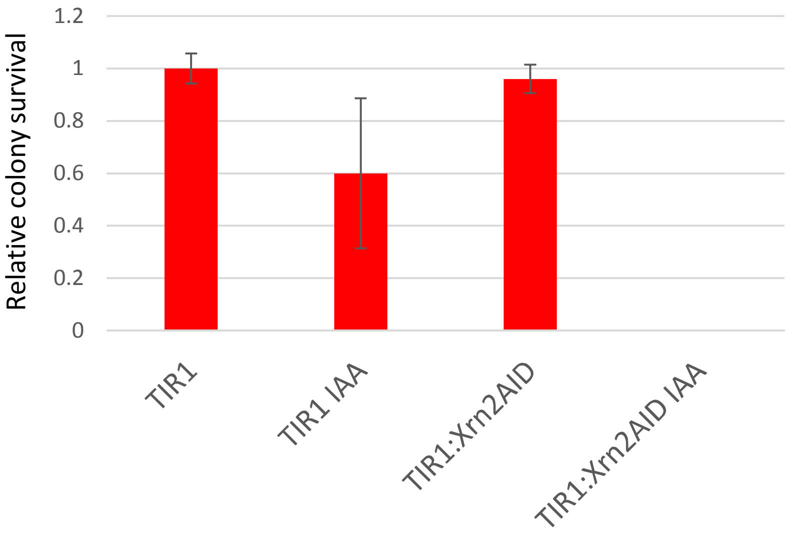

Eaton_Supplementary Fig2

A

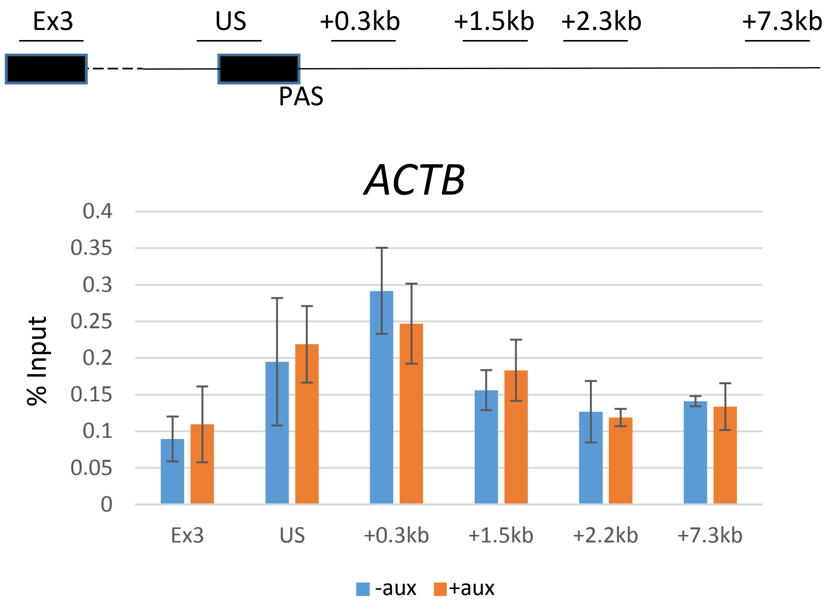

B

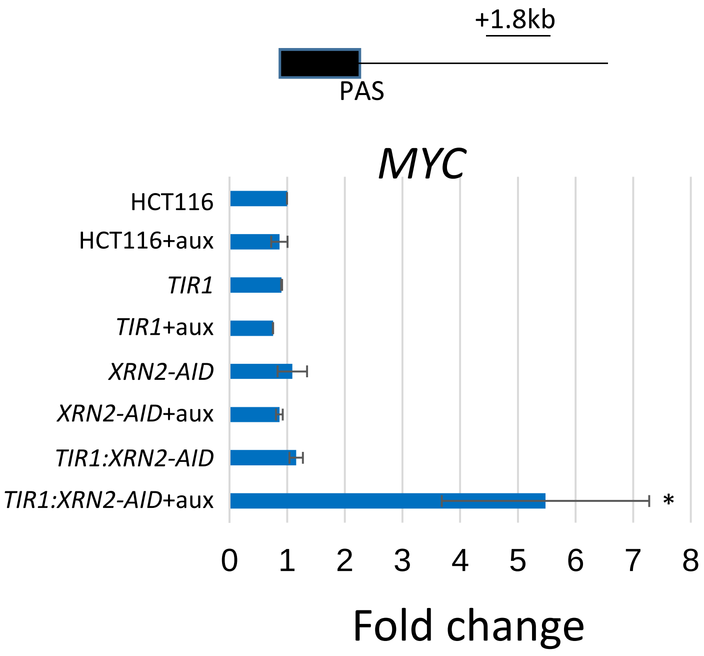

HCT116: unmodified
Tir1: Tir1 only
Xrn2-AID: tagged Xrn2 only
TIR1:Xrn2-AID: tagged Xrn2 and Tir1

Eaton_Supplementary Fig3

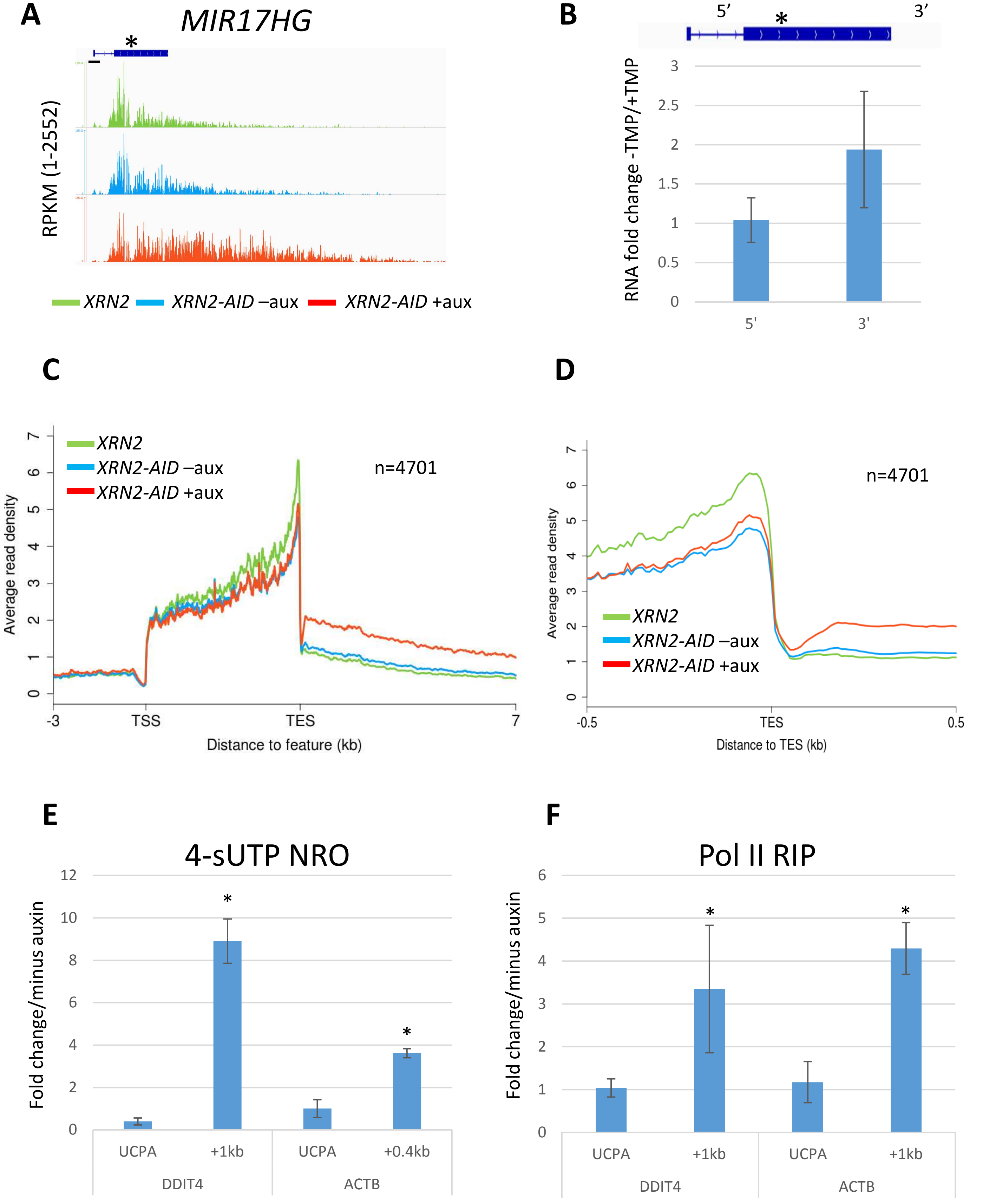

Eaton_Supplementary Fig4

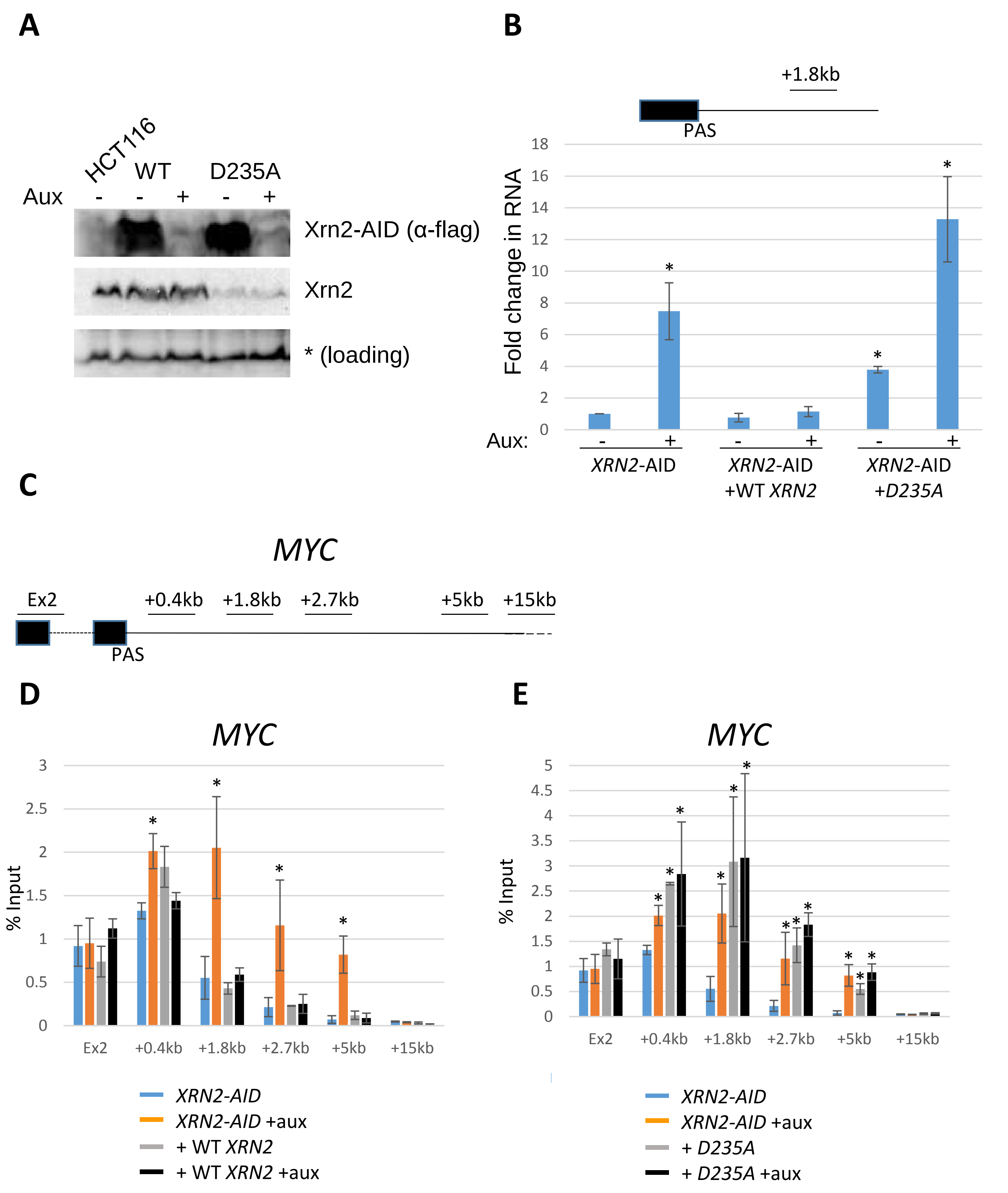

Eaton_Supplementary Fig5

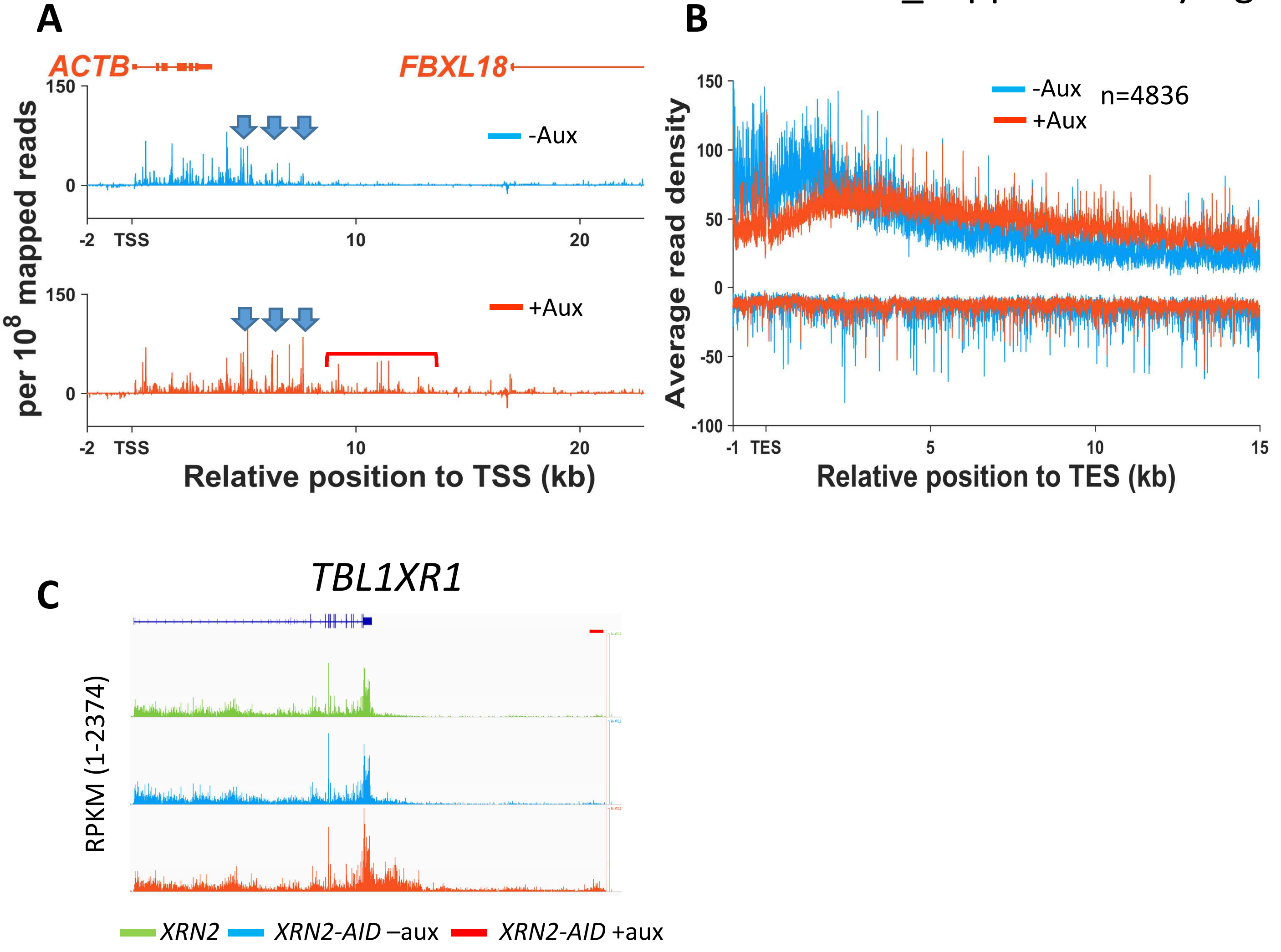

Eaton_Supplementary Fig6

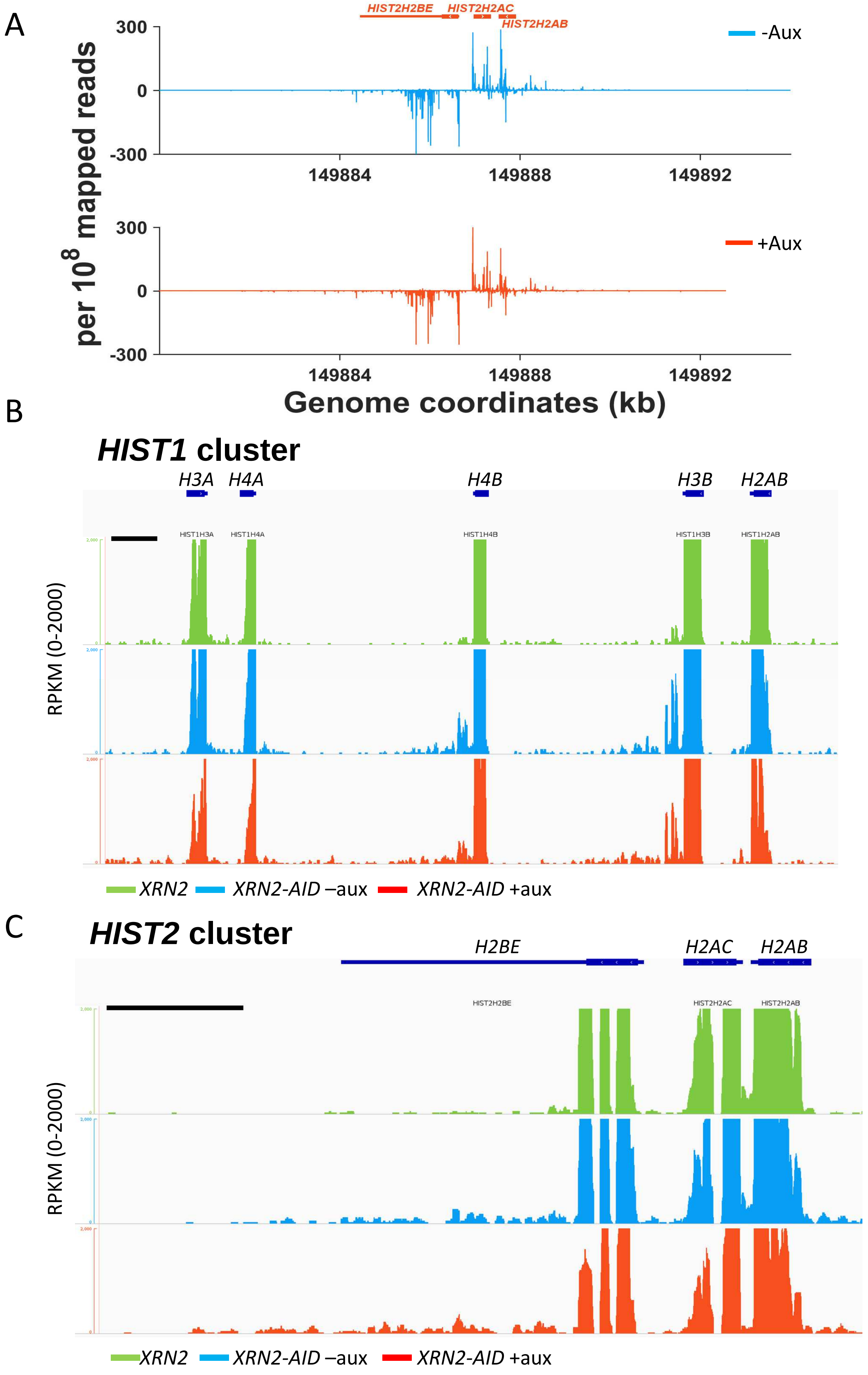

Eaton_Supplementary Fig7

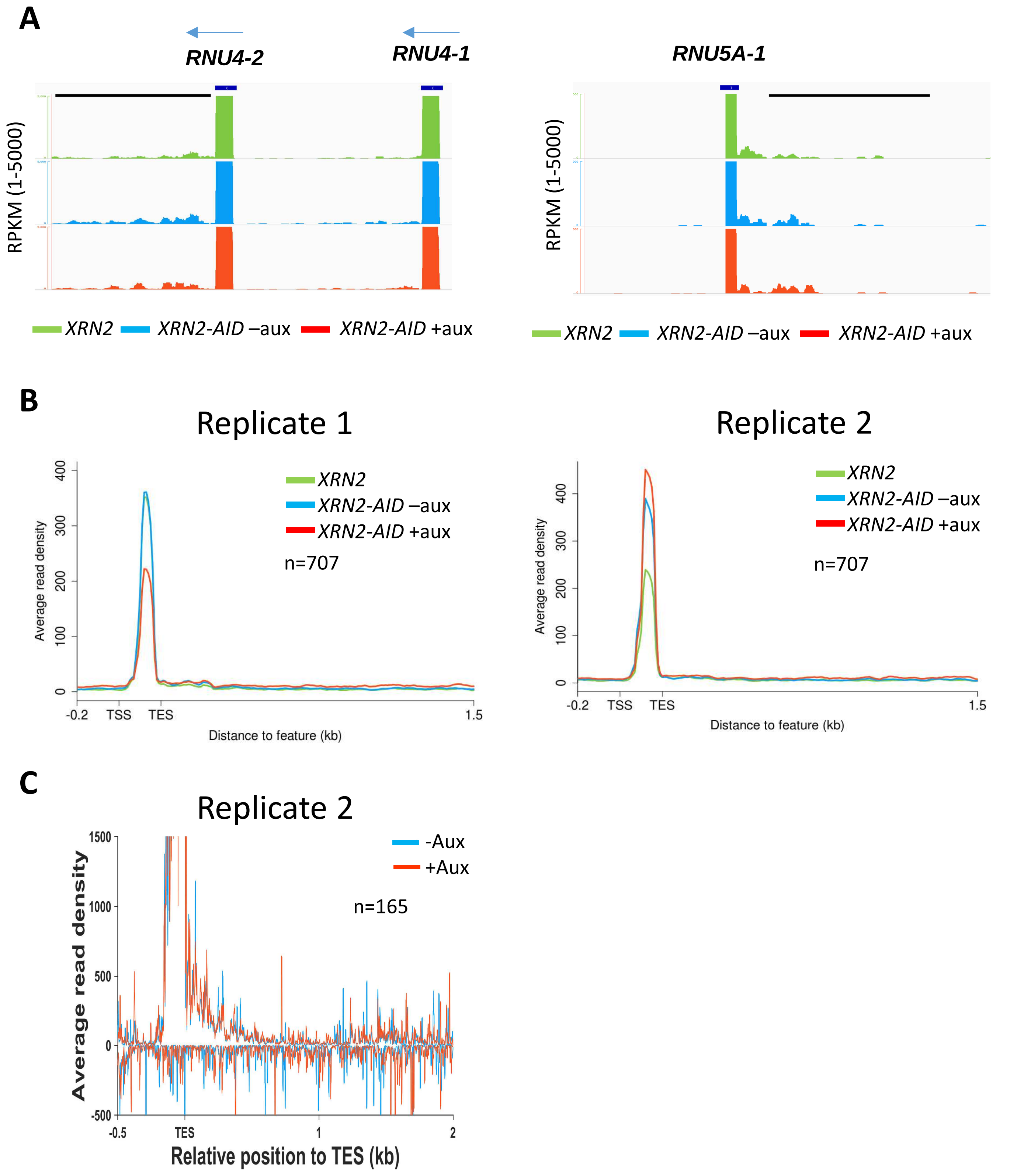

Eaton_Supplementary Fig8

A

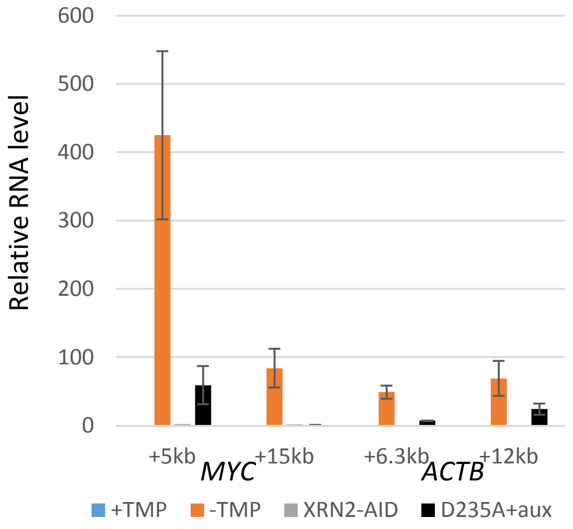

B

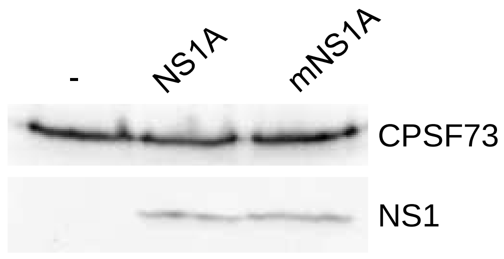

C

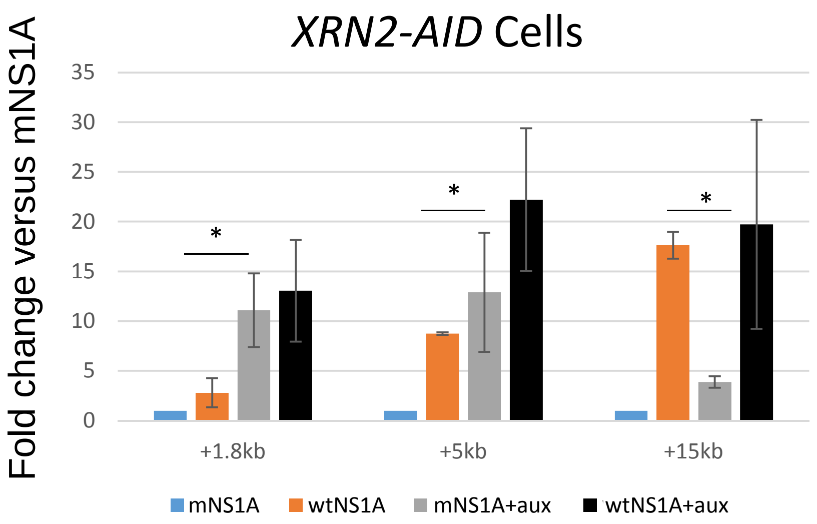

mNS1A: Xrn2 present, CPSF30 function normal
wtNS1A: Xrn2 present, CPSF30 function impaired
mNS1A+aux: Xrn2 absent, CPSF30 function normal
wtNS1A+aux: Xrn2 absent, CPSF30 function impaired

Eaton_Supplementary Fig9

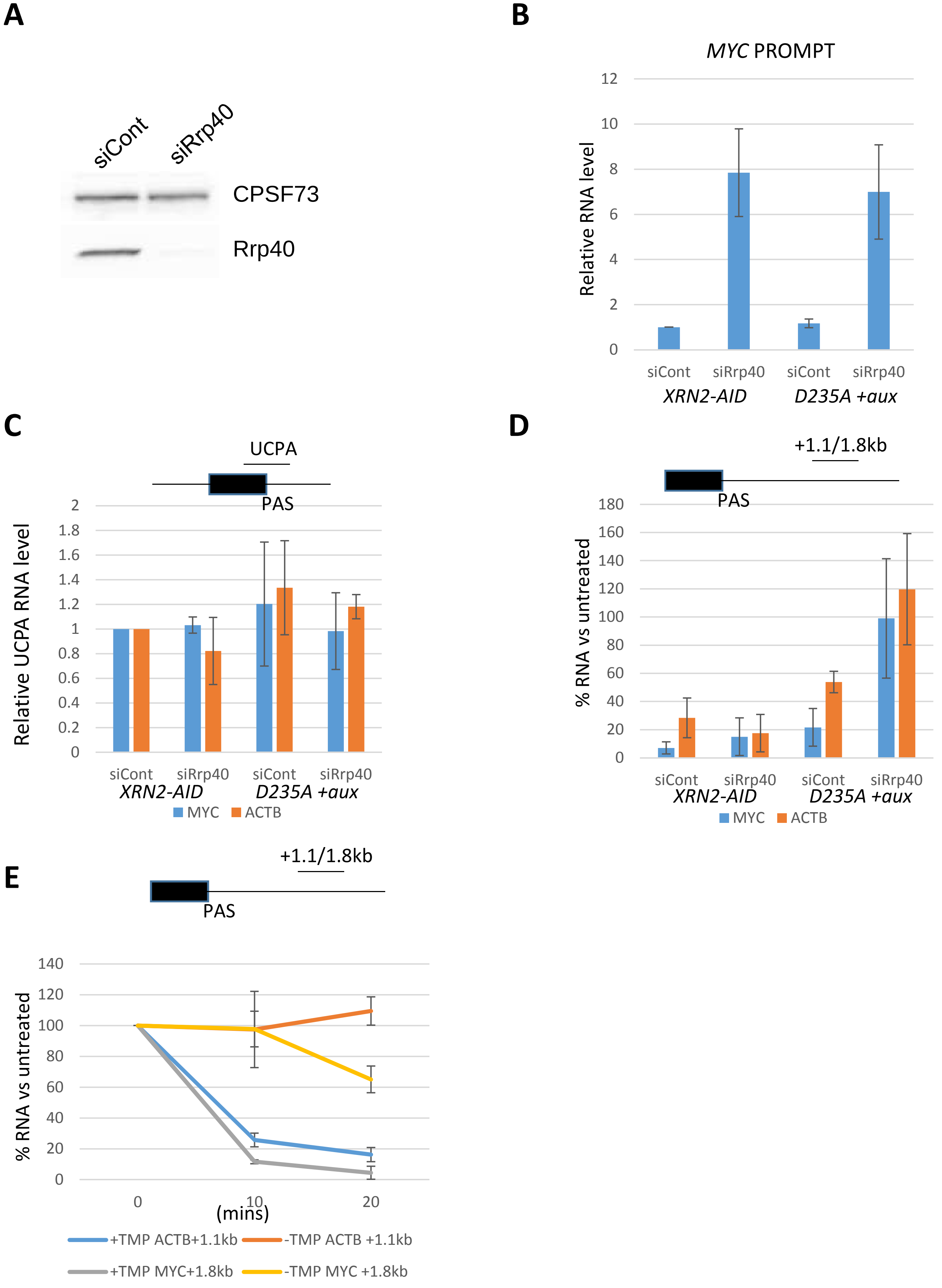

Supplementary Figure legends

FIGURE S1:

A. Representative growth curve of the indicated cell lines grown in the presence and absence of auxin. Note that only *TIR1:XRN2-AID* cells grown in the presence of auxin fail to multiply.

B. Colony formation assay for TIR1 or *TIR1:XRN2-AID* cells grown in the presence or absence of auxin. Auxin addition to *TIR1:XRN2-AID* cells completely prevents colony formation strongly suggesting that Xrn2 is essential. Importantly, tagging Xrn2 with AID causes no colony formation defect as no significant difference is observed between *TIR1* and *TIR1:XRN2-AID* cells grown in the absence of auxin. Graph shows results represented as a proportion of colonies relative to *TIR1:XRN2-AID* cells grown in the absence of auxin.

All error bars show standard deviation from at least three independent experiments.

FIGURE S2:

A. Pcf11 ChIP analysis on *ACTB* in *XRN2-AID* cells grown in the presence or absence of auxin. Values are expressed at % Input. Note that loss of Xrn2 does not affect the level or pattern of Pcf11 recruitment for this gene. Two Pcf11 antibodies were coupled to beads for this experiment.

B. qRT-PCR analysis of read-through RNA from +1.8kb downstream of the *MYC* PAS in unmodified HCT116 cells, HCT116 cells containing either *TIR1* only or *XRN2-AID* only or cells containing both Tir1 and Xrn2-AID. Note that auxin treatment only stabilises 3' flanking region RNA when both Tir1 and Xrn2-AID are present in the same cell. This experiment rules out any indirect effects of auxin, Tir1 or the AID tag. It also demonstrates that reduced levels of Xrn2-AID, compared to native Xrn2, do not impact on its ability to degrade 3' flanking region RNA as this species is present at similar levels in all cell lines in the absence of auxin. Asterisk shows $p<0.05$ versus unmodified HCT116 cells.

All error bars show standard deviation from at least three independent experiments.

FIGURE S3:

A. Nuclear RNA-seq trace of *MIR17HG* in samples obtained from *XRN2* unmodified cells and *XRN2-AID* cells treated or not with auxin. Site of miRNA cleavage is shown by an asterisk. Scale bar shows 1kb.

B. Analysis of the effect of CPSF73 depletion on levels of *MIR17HG* RNA from beyond the final AATAAA sequence in the ENSEMBL annotated gene (ENSG00000215417). Levels of RNA detected from this amplicon (3') are plotted alongside those obtained from an amplicon upstream (5') of the annotated Drosha cleavage site after normalisation to the level of spliced *ACTB* RNA. CPSF73 elimination has little impact on the level of flanking region RNA with levels raised by less than two fold. By contrast, 3' flanking region RNA from protein-coding genes (*MYC* and *ACTB*) is enhanced by over 10 fold. Thus, and as previously proposed, Drosha cleavage and not CPSF73 activity likely generates the Xrn2 substrates in this case. Details of the system for depleting CPSF73 can be found in main text Figure 5.

C. Biological repeat of metagene plot from Figure 2D obtained via nuclear RNA-seq on *XRN2* unmodified cells and cells treated or not with auxin. Graph shows region from 3kb upstream of the transcription start site (TSS) up to 7kb beyond the PAS (denoted as transcript end site, or TSS).

D. Biological replicate of metagene plot from Figure 2E.

E. 4sUTP NRO analysis of UCPA and 3' flanking RNA from *ACTB* and *DDIT4* in cells depleted or not of Xrn2-AID (1 hour auxin treatment). Quantitation is shown for +auxin samples relative to minus auxin after normalising to the level of unspliced *MYC* RNA. Asterisks denotes differences between + and – auxin where $p<0.05$.

F. Pol II RIP analysis of UCPA from *ACTB* and *DDIT4* in cells depleted or not of Xrn2-AID (1 hour auxin treatment). Quantitation is shown for +auxin samples expressed relative to minus auxin after normalising to the level of unspliced *MYC* RNA. Asterisks denotes differences between + and – auxin where $p<0.05$.

FIGURE S4:

A. Western blot of WT or D235A Xrn2 rescue lines and unmodified HCT116 cells. Top panel shows Xrn2-AID detected by anti-flag and its depletion by auxin in both WT and D235A lines. Xrn2 derived from integrated WT or D235A Xrn2 is shown in the middle panel. WT levels closely match those of native Xrn2 in unmodified HCT116 cells. A non-specific band (*) acts as a control for equal loading. D235A is expressed at a lower level possibly due to the deleterious effects of this inactive version.

B. qRT-PCR analysis of 3' flanking region total RNA (+1.8kb) from *MYC* in original *XRN2-AID* cells and those containing WT or D235A derivatives with samples collected in the presence or absence of auxin (1 hour). Values plotted relative to those obtained in *XRN2-AID* cells in the absence of auxin after normalising to the level of unspliced *MYC* RNA. Asterisks denote $p<0.05$ for differences compared to *XRN2-AID* cells minus auxin.

C. Diagram depicting the 3' end of *MYC* showing the positions of primer pairs used for ChIP analysis (not to scale).

D. *MYC* Pol II ChIP performed in *XRN2-AID* or cells expressing added back WT *XRN2* grown in the presence or absence of auxin (1 hour). Graph shows values expressed as a percentage of input. Asterisks denote differences where $p<0.05$ compared to *XRN2-AID* samples minus auxin.

E. *MYC* Pol II ChIP performed in *XRN2-AID* cells or cells expressing added back D235A Xrn2 grown in the presence or absence of auxin (1 hour). Graph shows values expressed as a percentage of input. Asterisks denote differences as compared to *XRN2-AID* samples minus auxin where $p<0.05$.

All error bars show standard deviation from at least three independent experiments.

FIGURE S5:

A. Example of *ACTB* mNET-seq tracks from *XRN2-AID* cells treated or not with auxin. Y-axis shows reads per 10^8 mapped reads. Xrn2 elimination causes read-through transcription evidenced by higher (blue arrows) and more extensive (red bracket) 3' flanking region signal.

B. Biological repeat of the metagene analysis of Xrn2 effect on global transcription termination on protein-coding genes as shown in Figure 3C.

C. Nuclear RNA-seq trace of *TBL1XR1* in samples obtained from *XRN2* unmodified cells and *XRN2-AID* cells treated or not with auxin. Scale bar shows 5kb.

FIGURE S6:

A. mNET-seq profiles over Histone H2 cluster region in *XRN2-AID* cells treated or not with auxin. Y-axes show signals per 10^8 mapped reads. It should be noted that reads below zero represent examples of Histone genes expressed on the opposite strand. The data show no Xrn2 effect on Histone gene transcriptional termination.

B. RNA-seq read coverage over Histone H1 cluster. Signals were obtained from *XRN2* unmodified cells and *XRN2-AID* cells treated or not with auxin. There is a very minor effect on read-through in untreated *XRN2-AID* cells versus *XRN2* unmodified cells but, as with the mNET-seq, there is no auxin dependent effect on transcriptional read-through. These data further argue that there is little involvement of Xrn2 in Histone gene transcriptional termination. Y-axes are RPKM.

C. As for B, but for Histone H2 genes.

FIGURE S7:

A. Examples of U snRNA gene RNA-seq traces from *XRN2* unmodified cells and *XRN2-AID* cells treated or not with auxin. There is no apparent effect of Xrn2 on levels of read-through RNA. Y-axes show RPKM.

B. Metagene analyses of snRNA gene RNA-seq repeats from *XRN2* unmodified cells and *XRN2-AID* cells treated or not with auxin. There is variation in read coverage over the gene body in each biological replicate potentially because short capped RNAs were not enriched for in the analysis. Nevertheless, there is no evidence in either replicate for an Xrn2 effect on the level of read-through RNA. Y-axes show RPKM.

C. mNET-seq metagene analyses of snRNA genes from an additional biological replicate of *XRN2-AID* cells treated or not with auxin. The Y-axes show average read density and are scaled to zoom into the termination region where signals are much lower than the highly expressed snRNA gene body. As with the RNA-seq, there is a lack of effect of Xrn2 loss on the termination of snRNA gene transcription.

FIGURE S8:

A. qRT-PCR analysis of extended read-through RNA from *MYC* and *ACTB* in *CPSF73-DHFR* cells grown in the presence and absence of TMP, *XRN2-AID* cells grown in the absence of auxin or *D235A* cells grown in the presence of auxin. For each amplicon, values are plotted relative to CPSF73-DHFR –TMP after normalisation to unspliced RNA from each gene. Note that in this experiment TMP removal has a greater effect on +5kb levels than in main text Figure 7. However, it is important to note that this experiment is on total RNA and was not subject to empty vector transfection and puromycin selection as was necessary for the experiment in main text Figure 7.

B. Western blot of *XRN2-AID* cells transfected with NS1A, mNS1A or, as a control, mock transfected. Specificity of NS1A signal is demonstrated by its absence in mock samples. CPSF73 was used to show equal loading.

C. qRT-PCR analysis of *MYC* transcriptional read-through in *XRN2-AID* cells transfected with NS1A or mNS1A and treated or not with auxin. Values are expressed as fold change over mNS1A transfected cells grown without auxin. Note that NS1A transfection results in more extensive read-through than Xrn2 depletion at +15kb even though the latter shows a stronger effect at upstream positions (+1.8kb and +5kb). This further supports that interference with PAS processing produces more profound read-through than Xrn2 loss. Asterisks show $p<0.05$ comparing loss of Xrn2 in the presence of mutant NS1A (where CPSF30 is unaffected) with inhibition of CPSF30 (via expression of NS1A). The accompanying text summarizes the 4 conditions tested.

All error bars show standard deviation from at least three independent experiments.

FIGURE S9:

- **A.** Western blot showing successful knock-down of hRrp40 in *XRN2-AID* cells (lower panel). Top panel shows CPSF73 used as a loading control.
- **B.** qRT-PCR analysis of *MYC* PROMPT, a *bone fide* exosome substrate, in *XRN2-AID* and auxin-treated *D235A* cells treated with control or hRrp40-specific siRNAs. Levels are expressed relative to those in control siRNA treated *XRN2-AID* cells.
- **C.** qRT-PCR analysis of non-PAS cleaved *MYC* and *ACTB* transcripts *XRN2-AID* and auxin-treated *D235A* cells treated with control or hRrp40-specific siRNAs. Levels are expressed relative to those in control siRNA treated *XRN2-AID* cells.
- **D.** qRT-PCR analysis of *ACTB* and *MYC* 3' flanking region RNA in control or hRrp40 siRNA treated *XRN2-AID* cells or *D235A* cells treated with auxin (all auxin for 1 hour) followed transcriptional inhibition by Flavopiridol (15 mins). RNA levels are expressed as a percentage remaining compared to t0.
- **E.** Act D time-course analysis of 3' flanking region transcripts from *MYC* and *ACTB* in *CPSF73-DHFR* cells grown in the presence or absence of TMP. Values in each time course are plotted as a percentage relative to amounts recovered at time zero.

All error bars show standard deviation from at least three independent experiments.

Supplemental Information

Plasmids

px330 (Addgene: 42230; (Cong et al. 2013)), pBABE osTIR1 (Addgene: (Holland et al. 2012)), psbi-blast (Addgene: 60526; (Kowarz et al. 2015)), psbi-puro (Addgene: 60523; (Kowarz et al. 2015)), pCMV(CAT)T7-SB100 (Addgene: 34879; (Mates et al. 2009)). For stable expression of Tir1, osTIR1-9xmyc was isolated from pBABE osTIR1 and cloned into psbi-blast using SfiI restriction sites. The repair templates for Xrn2 and CPSF73 gene editing were made using ~400bp homology arms. AID and DHFR tags were synthesised by Integrated DNA Technologies. WT and D235A Xrn2 vectors for stable transfection were made by insertion of the Xrn2 coding sequence into psbi-puro. CPSF73 plasmids for transient transfection were constructed by inserting a synthetic CPSF73 coding sequence into pcDNA5/FRT/TO. Sequences of all synthesised DNAs are provided below. A puromycin gene was then inserted 3' of CPSF73 with both genes separated by a T2A site. wtNS1A and mNS1A expression plasmids are described previously (Davidson et al. 2014).

Oligonucleotides:

ACTB Ex3: gcatgggtcagaaggattcc/ccacacgcagctcattgtag
ACTB US: tcaaggtgggtgtctttcct/cctgcttgctgatccacatc
ACTB UCPA: gcttttggtctccctggga/ctgcactctgggtaaggaca
ACTB +300: ctggcccttctatgtctccc/actcccaggaaatgcaggtg
ACTB +1.1kb: tgccttccctctgctagaag/tgtgcacagttgagagtcca
ACTB +1.7kb: ccaaccagatgtgttccgtg/caagaccaccaccacaatcg
ACTB +2.9kb: agaggaagagggccagaaac/tgcagtgacacaatcttggc
ACTB +6.3kb: aggaggcaatgctggagaat/gtacctgggaactctgcact
ACTB +12kb: cagggaagacgtgctaggaa/tcctttctcctctgctcagc
Myc Ex2: ggacgacgagaccttcatca/cgttgagagggtaggggaag
Myc US: attacaggtgtgagccaggg/agcctgcctcttttccaca
Myc UCPA: atcattgagccaaatcttaagttgtg/ctctgaaggggcaattgatga
Myc +400: aggcataaggactggggagt/tctggggtttgcgagataac
Myc +1.8kb: ggcgctcttaaacagctcag/ccaagctccacatccctaaa
Myc +2.7kb: agttttcacaatcccagcct/aatgctacaggggcccttag
Myc +5kb: tggaagaggagccaaaggag/ggaagctgcgttcatgtgat
Myc +15kb: tgggaaaggggcagttgta/atggtggggcattctctgaa
Myc PROMPT: gctggaaacttgttttaagg/tactggcagcagagatcat
DDIT4 US: ctggtgagtgtcccttctgt/taggcatggtgaggacagac
DDIT4 UCPA: agagttgagctggcaggg/gcacccatctccctcctttt
DDIT4 +1kb: agccctcacacccattttct/taggctgggagtggacaaag
MIR17HG 5': tcctgctagtattgctcgact/aggtccacgtgtatgactgg
MIR17HG 3': atgttctgaccagccctcaa/ttggtatgcactgagggtcc

siRNAs:
Control: silencer select siRNA negative control
hRrp40 siRNA: Thermo Fisher Silencer Select siRNA inventory#: s532991
siRNA transfection was performed using Lipofectamine RNAi MAX (Life Technologies).

Cell lines:

3xflag 3xmini-aid:
GGGGGTGGCAGCGGCGACTACAAAGATCACGACGGAGACTATAAAGATCACGACATC
GATTATAAAGATGACGACGATAAAGGTTCCGGTAAGGAAAAGAGCGCTTGCCCGAAGG
ATCCCGCAAAGCCCCCTGCTAAGGCTCAGGTGGTCGGTTGGCCACCTGTACGATCCTA
TCGAAAGAATGTCATGGTATCTTGCCAGAAGTCTTCCGGTGGTCCAGAGGCCGCTGCA
TTCGTAAAGGTTAGCATGGATGGTGCCCCTTATCTCCGGAAGATAGACTTGAGGATGTA
TAAGGGCGGCGGTAGCGGTGGTGGAAAAGAGAAATCCGCTTGCCCCAAGGATCCAGC
AAAACCTCCGGCCAAGGCTCAAGTGGTGGGTTGGCCCCCAGTAAGGTCTTACCGCAAA
AACGTCATGGTCAGCTGTCAAAAAAGTTCCGGCGGTCCAGAAGCAGCAGCATTCGTAA
AAGTCTCCATGGATGGGGCCCCCTATCTCAGAAAAATAGACCTGAGGATGTATAAAGGT
GGCGGATCAGGTGGGAAGGAGAAGTCCGCCTGCCCGAAGGACCCGGCCAAGCCACC
GGCGAAAGCGCAAGTGGTAGGTTGGCCTCCAGTTAGGAGCTATCGGAAAAATGTTATG
GTGAGTTGCCAGAAATCATCTGGAGGACCTGAAGCGGCTGCGTTTGTAAAGGTCTCTA
TGGACGGTGCGCCGTATTTGCGCAAGATCGATCTTAGAATGTATAAG

3xHA DHFR:
GGAGGCGGTTACCCATACGATGTTCCTGACTATGCGGGCTATCCCTATGACGTCCCGG
ACTATGCAGGATCCTATCCATATGACGTTCCAGATTACGCTGATATCATGATCAGCCTG
ATTGCTGCCCTTGCAGTGGACTATGTGATCGGTATGGAAAATGCTATGCCGTGGAACTT
GCCTGCTGACCTGGCCTGGTTTAAGAGGAACACGTTGAACAAGCCGGTTATTATGGGC
AGGCATACATGGGAGTCCATAGGGAGGCCCTTGCCAGGTCGAAAGAATATCATCCTTA
GTTCCCAGCCGTCTACGGACGACCGCGTTACCTGGGTTAAGAGCGTGGATGAGGCCAT

CGCGGCCTGTGGGGATGTTCCAGAAATAATGGTGATAGGGGGGGGTCGAGTAATAGA
ACAGTTTCTGCCTAAAGCCCAGAAGTTGTATCTTACTCATATAGATGCCGAAGTCGAAG
GTGATACACACTTCCCGGACTACGAGCCCGACGACTGGGAATCTGTATTCTCTGAATTC
CACGATGCTGACGCGCAGAATTCTCATTCTTACTGCTTCGAGATACTTGAACGCCGAGG

P2A nucleotide sequence:
GGATCAGGGGCCACTAACTTTTCCCTGCTGAAGCAGGCCGGAGACGTGGAGGAGAAC
CCCGGGCCC

Neomycin resistance gene:
ATGCCTGTAATTTCTACCCAGACTGGACGGGCCATGATTGAGCAAGACGGGCTCCACG
CTGGCAGCCCCGCAGCTTGGGTCGAGCGACTGTTCGGGTACGATTGGGCACAGCAGA
CAATAGGGTGCAGCGATGCCGCCGTCTTCCGGCTCAGCGCGCAAGGCCGGCCTGTCC
TGTTTGTTAAAACCGATCTGAGCGGGGCCCTGAACGAACTGCAGGATGAGGCGGCTAG
ACTTAGCTGGCTTGCGACCACCGGAGTGCCGTGTGCTGCCGTTCTGGACGTCGTAACA
GAGGCGGGAAGGGATTGGCTGCTGCTCGGGGAGGTCCCTGGCCAAGATTTGTTGTCC
TCCCACCTGGCACCTGCAGAGAAGGTAAGCATCATGGCAGATGCCATGCGCAGGCTG
CACACCCTGGATCCCGCCACGTGTCCTTTCGACCACCAGGCCAAGCACCGAATTGAGA
GGGCCAGGACACGCATGGAGGCCGGCCTGGTGGATCAGGACGATCTTGACGAGGAAC
ATCAGGGCCTCGCCCCAGCGGAGCTCTTTGCTCGGCTGAAAGCTAGAATGCCTGATGG
TGAAGATCTCGTCGTGACCCACGGAGATGCCTGCCTGCCCAACATCATGGTAGAAAAC
GGACGCTTCTCTGGCTTTATCGATTGTGGCCGGCTTGGAGTTGCTGATAGATATCAGGA
CATTGCACTCGCGACAAGAGACATTGCCGAGGAACTCGGTGGTGAATGGGCAGACCG
GTTCCTGGTGCTGTACGGGATCGCTGCCCCTGACTCACAGAGGATCGCATTTTACAGG
TTGCTGGACGAATTTTTTTAA

Hygromycin resistance gene:
ATGAAAAAGCCTGAACTCACCGCGACGTCTGTCGAGAAGTTTCTGATCGAAAAGTTCGA
CAGCGTCTCCGACCTGATGCAGCTCTCGGAGGGCGAAGAATCTCGTGCTTTCAGCTTC
GATGTAGGAGGGCGTGGATATGTCCTGCGGGTAAATAGCTGCGCCGATGGTTTCTACA
AAGATCGTTATGTTTATCGGCACTTTGCATCGGCCGCGCTCCCGATTCCGGAAGTGCTT
GACATTGGGGAGTTCAGCGAGAGCCTGACCTATTGCATCTCCCGCCGTGCACAGGGTG
TCACGTTGCAAGACCTGCCTGAAACCGAACTGCCCGCTGTTCTTCAGCCGGTCGCGGA
GGCTATGGATGCGATCGCTGCGGCCGATCTTAGCCAGACGAGCGGGTTCGGCCCATT
CGGACCGCAAGGAATCGGTCAATACACTACATGGCGTGATTTCATATGCGCGATTGCT
GATCCCCATGTGTATCACTGGCAAACTGTGATGGACGACACCGTCAGTGCGTCCGTCG
CGCAGGCTCTCGATGAGCTGATGCTTTGGGCCGAGGACTGCCCCGAAGTCCGGCACC
TCGTGCACGCGGATTTCGGCTCCAACAATGTCCTGACGGACAATGGCCGCATAACAGC
GGTCATTGACTGGAGCGAGGCGATGTTCGGGGATTCCCAATACGAGGTCGCCAACATC
TTCTTCTGGAGGCCGTGGTTGGCTTGTATGGAGCAGCAGACGCGCTACTTCGAGCGGA
GGCATCCGGAGCTTGCAGGATCGCCACGCCTCCGGGCGTATATGCTCCGCATTGGTCT
TGACCAACTCTATCAGAGCTTGGTTGACGGCAATTTCGATGATGCAGCTTGGGCGCAG
GGTCGATGCGACGCAATCGTCCGATCCGGAGCCGGGACTGTCGGGCGTACACAAATC
GCCCGCAGAAGCGCGGCCGTCTGGACCGATGGCTGTGTAGAAGTACTCGCCGATAGT
GGAAACCGACGCCCCAGCACTCGTCCGAGGGCAAAGGAATAG

SV40 poly(A) signal:
Aacttgtttattgcagcttataatggttacaaataaagcaatagcatcacaaatttcacaaataaagcatttttttcactgcattctagtt
gtggtttgtccaaactcatcaatgtatctta

Xrn2 homology arms
5’:
GTGTAATAAGTCTAAATTGATGTGGGTATCTTACCACAAAGTGACTTGAATTACTACTGC
TAGGACAGTGAGAAAATTGAGAACCACTGTCTGTACATGTTGTTTACACAGAACACTTTA
GTTATTTGTGTGCATTTGTGATTGTTAAGGTTTTTTGTTTTATTTTTCAGTAATAGCATTTG

TGCTAGCCTCCAACTTTGCAACAAGTCTGTATTAAAGCTCTGGATCAAAGCACCTTTTAT
GGGGCCTTTCCATGTGCTGTACCTTTAACACATACTCAGTTTCCTTATGATGTGTTTTTC
CATAGAGGTTTAAAGTTAACTGACTTGCAGGAGTATCGGTCCAGAAAATAAACTCTTTCT
TTTGTTTATTTTCAGGGATATCCCAGAGAAGGAAGAAAATACCCTTTGCCACCACCCTCA
GGAAGATACAATTGGAAT

3':
GCTTTTGTAAAGCTTTCCCAAATCCTTTCATCATTCTACAGTTTTATGCTATTTGTGGAAA
GATTTCTTTCTCAAGTAGTAGTTTTTAATAAAACTACAGTACTTTGTGTATTTCTTTTAACT
GTGTATATTTCTACTGATCTGATCTCACTGTTTATGTTGCTTTCCAAAGATGTATGTTGCA
TAATACAGTGGATCTGAATTTATTATTGCTTATAAAACACATTTGATGGAATAGGAGTACT
GGTTTTTCATAATGGTTAAAAATGAAACCAGCTGTGGATTTCAAAACACAGTGTATTCTA
GATCATCTAAGATCCATGCTGATTTTTATTGCACAAGAATTAGGTTTGAACTCGAGCTGG
AACCTCAGCAAACTAGAGTATAT

Xrn2 guide RNA target: AGGGATATCCCAGAGAAGGA

CPSF73 homology arms
5':
CCACATCCATTCCTTGCCAAGTATCATTTACTAGATCAAACTGTGGGCTTTGATGTAAAT
GTAGTTTACTAGACTTTCCCCAGTCTTTCACCCCAGCCTCAAGTCATCACTAATTAGGAC
CGTGCTGCTGTCAGGAAGCACTGCACGCCCACAAGTGTGTAGGGCGGCCGTTCTGTTT
CATGGTAATCAGTCCCACCATGACCTCTGCACACACAGATGATTGTTCTTTTTTTTAGTT
TGAGACCCGGTCTCGCAGTGCCGCCCAGGCTGGAGTGCAGTGGTGCAGTCACAGCTC
ACTGCAGCCTCAACCTTCCCGGCTCAGTGATCCTCCCACCTCAGACTCTTATCTGGGAC
CACAGGCACACGCCACCACAGCTGGCTAATTTTTTATGAGATGATGGTTTTTTTAAAGA
GTATTCATTTATCTTCTATATAATCATTATAGACTTAATTCTAACAGTCTTGTTTGTGCCTC
ACTTTCAGACTGTAGAATGTGAAGAGGGAAGTGAAGACGATGAATCCCTCCGAGAAAT
GGTGGAGCTGGCTGCACAGAGACTGTACGAAGCCCTGACGCCAGTTCAC

3':
GACTGTGCCTGTATATGAACTTTGAAAAAATACTTGACTCTACTTTTGTTACCTAAAATAA
AATGCATTCGTTTCTCTGGGGGAGCCTGTTTACTTTTAATGTCAAATGGCCTTTATTTCA
ACAGCCTGAATACTGCTAAATTGCTAATTAATTTGTCCATTATTCTAGAACTAACTACTAG
ATCAACTGCCCATTATTTTAGAATTTTGGATTCTTCTTCCAGGCATGTATGTGCAGCTCC
CATTGAAACCATCAAGATCTGCCGATAGCAACCGCTGCTGGTTACCCTCTCCTCTGGG
GTAACCAATTTGAGTTAATAATAAGGATTCTAAGTTGCACTTGAATCTTTTCTGTCTTCAT
CTCCACTGCTGCTGTTCGAGTCCAAGTCTACTCTCCCCTCTGAATTCCTGCAACCACCT
CCATCTCCTCCCCTATAGCTGATTCCTGGAACAGACCTGGCCTC

CPSF73 guide RNA target: GGCTGCACAGAGACTGTACG

Synthesised CPSF73 sequence:

ATGAGCGCAATTCCGGCGGAAGAGAGTGACCAACTTTTGATCCGCCCTCTGGGGGCAG
GACAAGAAGTTGGACGCTCCTGTATTATATTGGAGTTCAAAGGCCGCAAAATAATGTTG
GACTGCGGAATCCACCCAGGATTGGAGGGAATGGACGCACTGCCCTATATCGATCTCA
TAGATCCCGCCGAGATCGATCTCCTGCTCATTAGCCACTTTCACCTGGACCACTGTGGC
GCACTGCCATGGTTCCTTCAGAAAACATCATTTAAGGGCCGGACGTTTATGACCCACGC
GACGAAGGCTATCTACAGGTGGCTTTTGAGCGACTACGTTAAGGTTTCAAACATAAGCG
CCGATGACATGCTCTATACGGAAACGGATCTGGAAGAATCCATGGACAAGATAGAGAC
AATAAACTTTCATGGAGTTAAAGAAGTAGCGGGCATAAAATTCTGGTGCTATCACGCCG
GACACGTCTTGGGCGCAGCCATGTTTATGATAGAGATAGCGGGAGTCAAACTGCTGTA
TACTGGAGACTTCAGTCGGCAGGAGGATAGGCATTTGATGGCAGCAGAGATTCCAAAT

ATCAAGCCTGATATACTTATAATCGAATCCACGTACGGCACTCATATTCACGAAAAGCG
AGAGGAGCGAGAAGCTAGGTTCTGCAACACCGTGCATGACATTGTAAATAGAGGGGGC
CGGGGCCTCATTCCGGTATTCGCTCTGGGGAGAGCACAGGAGCTTCTGCTTATCCTTG
ACGAATACTGGCAAAATCACCCGGAGTTGCATGATATCCCAATATATTATGCCAGCAGT
TTGGCTAAAAAGTGCATGGCAGTATATCAGACATATGTCAACGCTATGAATGACAAGAT
ACGCAAGCAAATAAACATAAATAATCCATTTGTGTTCAAACACATCAGTAATCTGAAGAG
TATGGACCACTTTGACGATATTGGTCCTAGCGTAGTCATGGCTAGCCCAGGAATGATGC
AAAGCGGTCTGAGCAGAGAGCTGTTCGAGTCCTGGTGTACCGATAAAAGGAATGGTGT
TATTATCGCCGGGTATTGTGTAGAGGGGACCCTCGCAAAACACATTATGAGCGAGCCG
GAAGAAATAACTACTATGAGTGGCCAGAAATTGCCGTTGAAGATGTCTGTTGACTACAT
TTCTTTCTCAGCTCATACGGATTACCAGCAAACCTCTGAGTTCATCCGCGCATTGAAAC
CACCACATGTTATTCTTGTCCATGGCGAGCAAAATGAAATGGCACGGTTGAAGGCTGCA
TTGATCCGCGAATACGAAGACAATGATGAGGTTCACATCGAGGTCCACAATCCAAGGAA
TACTGAGGCAGTAACGCTTAACTTCCGCGGGGAAAAGCTGGCGAAAGTTATGGGCTTC
TTGGCGGATAAGAAACCGGAACAAGGCCAACGGGTTAGCGGCATACTGGTCAAGCGAA
ATTTCAATTATCACATCCTTAGCCCGTGCGATCTTAGCAATTACACTGATCTTGCCATGA
GTACCGTCAAGCAAACCCAAGCTATTCCTTATACAGGCCCCTTCAACCTGCTGTGTTAC
CAGCTTCAAAAGTTGACTGGAGATGTGGAGGAGCTGGAGATTCAAGAAAAGCCCGCAC
TTAAGGTCTTTAAAAATATCACGGTAATTCAAGAACCAGGAATGGTAGTGCTCGAATGG
CTCGCAAATCCTAGCAACGACATGTATGCTGATACCGTAACAACTGTAATACTCGAGGT
TCAGAGTAATCCAAAGATAAGGAAAGGAGCGGTACAGAAGGTCTCTAAGAAGCTTGAAA
TGCACGTGTACTCAAAACGACTTGAGATAATGCTGCAAGACATCTTCGGAGAGGACTGC
GTCAGTGTTAAAGATGACTCCATACTCAGTGTGACTGTAGATGGGAAGACAGCAAATCT
TAACCTGGAGACCAGAACGGTAGAGTGTGAGGAAGGAAGTGAAGACGACGAATCCTTG
AGGGAGATGGTCGAGCTCGCAGCCCAGCGGCTTTATGAAGCCTTGACCCCCGTACACT
AA

Supplemental References:

Cong L, Ran FA, Cox D, Lin S, Barretto R, Habib N, Hsu PD, Wu X, Jiang W, Marraffini LA et al. 2013. Multiplex genome engineering using CRISPR/Cas systems. *Science* **339**: 819-823.

Davidson L, Muniz L, West S. 2014. 3' end formation of pre-mRNA and phosphorylation of Ser2 on the RNA polymerase II CTD are reciprocally coupled in human cells. *Genes & development* **28**: 342-356.

Holland AJ, Fachinetti D, Han JS, Cleveland DW. 2012. Inducible, reversible system for the rapid and complete degradation of proteins in mammalian cells. *Proceedings of the National Academy of Sciences of the United States of America* **109**: E3350-3357.

Kowarz E, Loscher D, Marschalek R. 2015. Optimized Sleeping Beauty transposons rapidly generate stable transgenic cell lines. *Biotechnol J* **10**: 647-653.

Mates L, Chuah MK, Belay E, Jerchow B, Manoj N, Acosta-Sanchez A, Grzela DP, Schmitt A, Becker K, Matrai J et al. 2009. Molecular evolution of a novel hyperactive Sleeping Beauty transposase enables robust stable gene transfer in vertebrates. *Nature genetics* **41**: 753-761.
